# Investigating the Acute Effect of Different Training Protocols on Heart Rate Variability

**DOI:** 10.3390/sports14070299

**Published:** 2026-07-13

**Authors:** Burhan Demirkıran, Tuba Melekoğlu, Grzegorz Żurek

**Affiliations:** 1 Department of Movement & Training Sciences, Institute of Health Sciences, Akdeniz University, Antalya 07070, Türkiye; burhandemirkiran@yahoo.com; 2 Department of Coaching Education, Faculty of Sport Sciences, Akdeniz University, Antalya 07070, Türkiye; 3 Department of Biological Principles of Physical Activity, Wroclaw University of Health and Sport Sciences, 51612 Wroclaw, Poland; grzegorz.zurek@awf.wroc.pl

**Keywords:** autonomic nervous system, Greco-Roman wrestling, interval training, athlete monitoring

## Abstract

This study examined the acute effects of high-intensity interval training (HIIT) and prolonged endurance training (ET) on heart rate variability (HRV) in elite Greco-Roman wrestlers and assessed the usefulness of HRV for monitoring post-exercise recovery. Using a longitudinal, within-subject, repeated-measures design, 13 elite male wrestlers completed two training protocols—differing in intensity, duration, modality, and work–rest structure—in a fixed, non-randomized order (ET on Day 7, HIIT on Day 22), separated by a 15-day washout period. HRV was recorded at baseline, pre-exercise, during training, and 24 h post-exercise, and analyzed with a linear mixed model and Bonferroni-adjusted post hoc comparisons. A significant main effect of Timepoint was found for all HRV parameters (SDNN, RMSSD, LF/HF ratio, and the lnRMSSD-derived HRV Score), reflecting marked reductions during exercise and partial recovery by 24 h (e.g., SDNN decreased by approximately 73% in ET and 82% in HIIT). SDNN also differed significantly between training types overall, but heart rate did not differ between protocols during exercise (*p* = 0.127). No between-protocol difference for any HRV parameter survived Bonferroni correction at any timepoint, despite a large effect size for the HRV Score during exercise (d = 0.79); the corresponding effect for raw RMSSD, the primary time-domain outcome, was small (d = 0.36) and non-significant. Both protocols showed comparable recovery at 24 h. Because protocol order was fixed rather than randomized, these findings should be interpreted as a comparison between two specific field-based conditioning sessions rather than a controlled test of exercise intensity alone.

## 1. Introduction

Heart rate variability (HRV) refers to the fluctuations in the intervals between successive heartbeats, representing dynamic adjustments of the autonomic nervous system (ANS). Rather than being constant, the time between R-R intervals varies slightly, offering a non-invasive index of cardiac autonomic regulation. HRV analysis includes time-domain and frequency-domain measures, each providing insights into sympathetic and parasympathetic modulation. Among these, the standard deviation of normal R-R intervals (SDNN) and the root mean square of successive differences (RMSSD) are key time-domain indices, while high-frequency (HF) power represents parasympathetic activity in the frequency domain. Elevated SDNN values generally indicate enhanced autonomic function and cardiovascular adaptability [1,2].

The ANS plays a central role in regulating involuntary physiological processes such as heart rate, blood pressure, respiration, and digestion. HRV is therefore recognized as a marker of autonomic balance. The sympathetic branch, responsible for the “fight-or-flight” response, increases cardiovascular activity during stress, whereas the parasympathetic branch, associated with the “rest-and-digest” response, promotes recovery and energy conservation. A dynamic balance between these branches is essential for physiological homeostasis and athletic performance [3,4].

Numerous intrinsic and extrinsic factors modulate HRV, with physical exercise being a primary external influence. The autonomic response to exercise differs depending on the intensity, type, and duration of the activity. Low-intensity aerobic exercise typically enhances parasympathetic activity, thereby increasing HRV [5,6]. In contrast, high-intensity exercise induces sympathetic dominance, temporarily reducing HRV due to increased cardiovascular and metabolic demands. However, post-exercise parasympathetic reactivation can promote long-term autonomic adaptation and improved cardiovascular efficiency [7].

The post-exercise increase in HRV is likely associated with reduced sympathetic dominance and enhanced parasympathetic reactivation. HRV markers indicating high parasympathetic activity, which can be evaluated after exercise, provide valuable insights into recovery status. Previous studies have reported that elite athletes exhibit pronounced parasympathetic reactivation and adaptive changes in cardiac autonomic control, serving as indicators of efficient recovery following exercise [8,9,10]. The magnitude and duration of exercise-induced sympathetic activation and the degree of subsequent HRV suppression are typically modulated by the individual’s fitness level, stress resilience, and training experience [11,12].

While acute HRV suppression during exercise is expected, elite athletes often exhibit attenuated reductions in parameters such as SDNN and RMSSD during high training loads (e.g., HIIT or endurance exercise), in contrast to recreational athletes. This suggests a more efficient sympathetic response and greater physiological adaptability [13,14]. Moreover, HRV analysis during and after exercise provides valuable insights into training load, fatigue, and recovery dynamics and has therefore gained prominence as a practical performance monitoring tool in elite sport [15,16].

However, despite HRV’s growing use, its interpretation in elite athletes remains complex and sometimes contradictory. While increases in vagal-mediated HRV indices are generally associated with positive adaptations (e.g., improved fitness, autonomic balance), elite athletes often show inconsistent patterns. Studies have reported both increases and decreases in HRV accompanying signs of maladaptation, such as overreaching or stagnation in performance [17]. Paradoxically, improvements in cardiorespiratory fitness have also been observed alongside reductions in HRV possibly due to “HRV saturation,” a phenomenon in highly trained individuals where further improvements in autonomic efficiency no longer translate to higher HRV values [16].

This indicates that elite athletes possess a unique HRV fingerprint requiring individualized, longitudinal monitoring rather than reliance on isolated values. Daily or weekly fluctuations in HRV should be interpreted with caution, and meaningful trends should be extracted using appropriate statistical techniques such as rolling averages or baseline-corrected changes [13,16]. In this context, HRV becomes most useful not as a standalone diagnostic, but as part of a comprehensive athlete monitoring system tailored to each athlete’s physiological baseline and competitive demands [17].

Recent work with Olympic and World Champion athletes has shown that strategic use of HRV metrics, particularly indices less prone to saturation, can effectively track fitness (chronic adaptation) and freshness (acute readiness). This nuanced interpretation allows coaches and practitioners to align training load with recovery capacity, thereby reducing injury risk and optimizing performance outcomes during critical periods such as tapering or competition phases [13,16,18].

Greco-Roman wrestling is characterized by repeated high-intensity explosive efforts interspersed with brief recovery intervals, placing substantial demands on both aerobic and anaerobic energy systems. The intermittent, contact-based nature of wrestling—involving maximal exertion during bouts and short recovery windows between matches in tournament formats—makes autonomic recovery a critical determinant of performance capacity [19,20]. Given these demands, HRV monitoring has particular ecological relevance in wrestling, as it may provide coaches with real-time, non-invasive information about an athlete’s readiness for subsequent training or competition [20,21].

Within combat sports, HRV has been studied primarily in judo populations. Campos et al. [22] demonstrated that resting SDNN and RMSSD were significantly correlated with intermittent judo-specific performance, suggesting that higher parasympathetic tone is associated with better capacity for repeated high-intensity efforts. More recently, Güngör et al. [20] reported incomplete HRV recovery following simulated wrestling matches, with persistent autonomic suppression despite passive rest and active recovery interventions, underscoring the acute autonomic burden of wrestling-specific exertion. Furthermore, Kajaia et al. [21] observed that wrestlers were among the most frequently affected athletes in cases of non-functional overreaching and overtraining syndrome, accompanied by significant disruptions in HRV-based autonomic regulation. Despite this growing body of evidence in adjacent disciplines, the acute HRV responses to specific training modalities such as HIIT and endurance training have not been systematically examined in elite Greco-Roman wrestlers.

To address this gap, the present study investigates the immediate effects of HIIT and endurance exercise protocols on HRV in elite Greco-Roman wrestlers. A secondary aim is to evaluate HRV responses during the 24 h post-exercise recovery period. Ultimately, this study aims to contribute to the literature by exploring the potential of HRV as a physiological biomarker for guiding training load, assessing performance capacity, and enhancing recovery strategies in elite athletes. Based on previous literature demonstrating greater autonomic perturbation following high-intensity exercise, it was hypothesized that both protocols would reduce HRV during exercise, with the HIIT protocol producing a greater acute suppression of HRV indices than the ET protocol. A secondary hypothesis was that HRV would demonstrate partial recovery within 24 h following both exercise sessions.

## 2. Materials and Methods

### 2.1. Experimental Approach to the Problem

This longitudinal study employed a short-term repeated-measures design to investigate changes in HRV among elite Greco-Roman wrestlers from the Danish National Team, in response to different training modalities. Data collection took place in Nykøbing Falster, Denmark, at the national training camp held two months prior to international competitions.

Anthropometric assessments including body mass, height, and age were recorded on the first day of the camp. No dietary interventions were implemented, and as wrestling is a weight-classified sport, athletes’ weight fluctuations were closely monitored throughout the study. According to both athletes and coaches, no weight loss occurred during the camp or training sessions.

The study incorporated two distinct exercise protocols (ET and HIIT) separated by a 15-day washout period to minimize potential carryover effects. The protocol sequence was fixed in accordance with the structure of the national training camp: ET was performed on Day 7 and HIIT on Day 22. Randomization or counterbalancing was not feasible given the operational constraints of the elite training environment. Consequently, the present study should be considered a field-based repeated-measures investigation rather than a randomized crossover trial. Potential order effects—including cumulative fatigue, adaptation across the training camp, and other time-dependent changes—cannot be completely excluded.

To assess autonomic nervous system (ANS) modulation, HRV data were collected using validated parameters: the standard deviation of normal-to-normal RR intervals (SDNN), the root mean square of successive RR interval differences (RMSSD), and the low-frequency to high-frequency power ratio (LF/HF).

Measurements were performed under resting conditions for 5 min each morning, in a supine position immediately after waking up, to reduce the influence of confounding variables. HRV data were averaged over multiple time points:

- Baseline (BASE): At least one week prior to each training protocol.
- Pre-exercise (PRE): On the day of each training session, before exercise.
- During Exercise (TRAINING): Average HRV during the session.
- Post 24 h (POST): 24 h after each training session.

**Figure 1:**
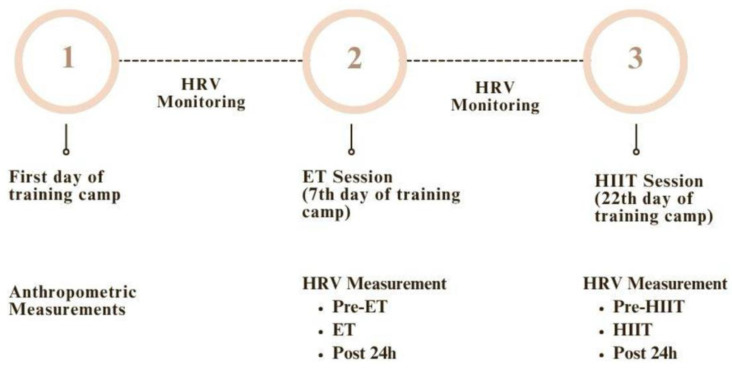
Overview of the Study Design and Timeline. Schematic representation of the experimental protocol used to evaluate acute heart rate variability (HRV) responses to endurance training (ET) and high-intensity interval training (HIIT) in elite Greco-Roman wrestlers. Daily HRV monitoring was conducted throughout the training camp. Key measurements were taken at four time points: baseline (BASE), immediately before training (PRE), during training (TRAINING), and 24 h post-exercise (POST 24 h). The timeline reflects the sequence of training interventions and HRV assessments used to compare autonomic responses and recovery profiles across training modalities.

Heart rate data, including maximal heart rate (HRmax), were also recorded. HRV values obtained during the training sessions were averaged to represent the overall within-session HRV response. Figure 1 illustrates the overall study design and timeline.

### 2.2. Subjects

Thirteen elite male wrestlers (mean age: 21.26 ± 1.34 years; body mass: 74.82 ± 8.01 kg; height: 177.07 ± 6.56 cm) from the Danish National Greco-Roman Wrestling Team voluntarily participated in this study. All athletes had a minimum of 10 years of competitive training experience at the national and international levels. Several participants had extensive international competition experience, including medal-winning performances at major continental and global championships.

All participants provided written informed consent prior to the study. Inclusion criteria were: (a) membership in the Danish National Greco-Roman Wrestling Team, (b) active participation in all scheduled training sessions, and (c) absence of injury or illness limiting training participation. Exclusion criteria included cardiovascular disease, use of medications known to affect autonomic function, and inability to complete both training protocols or HRV assessments. The research protocol adhered to the ethical principles outlined in the Declaration of Helsinki and was approved by the Akdeniz University Clinical Research Ethics Committee (TBAEK-194/30.01.2025). Additionally, institutional permission for data collection was obtained from the Danish Wrestling Federation.

A priori power analysis was conducted using G*Power (version 3.1.9.7; F tests: ANOVA, repeated measures, within factors) to determine the required sample size for detecting a medium-to-large interaction effect (f = 0.30) in a 2 (Training Type: ET vs. HIIT) × 4 (Timepoint: BASE, PRE, TRAINING, POST 24 h) within-subjects design, with α = 0.05, power (1 − β) = 0.80, an assumed correlation among repeated measures of r = 0.5, and a nonsphericity correction of ε = 1 (sphericity assumed). Under these assumptions, a minimum of 12 participants was required. The final sample of 13 elite wrestlers met this a priori target for the primary interaction effect.

### 2.3. Training Protocol

HRV measurements were conducted on Day 7 (ET) and Day 22 (HIIT) of the preparatory camp for international competitions. Both the endurance and interval training sessions were implemented at 10:00 AM on the final day of a one-week training block.

Both protocols were selected because cycling- and running-based conditioning are standard components of elite wrestling preparation, widely employed alongside mat-based technical training to develop the aerobic base and high-intensity work capacity required for repeated explosive efforts during competition [19]. Cross-training modalities such as cycling and interval running are preferred in national camp settings because they allow precise heart rate-based intensity control that is difficult to achieve during wrestling-specific technical sessions. Importantly, the protocols examined in this study were not designed as laboratory constructs but were actual training sessions implemented by the Danish national team coaching staff as part of the camp’s standard conditioning program. Therefore, although the protocols do not replicate mat-based wrestling movements, they represent the conditioning work genuinely performed by elite wrestlers in preparation for international competition.

The ET session was performed on an intercity cycling path in Denmark. During the session, athletes were followed by coaches in vehicles and received verbal instructions to either maintain or increase their cycling pace. The session concluded with athletes cycling at their maximal effort until exhaustion.

Both endurance training (ET) and high-intensity interval training (HIIT) sessions were preceded by a standardized 15 min traditional warm-up consisting of dynamic mobility drills and light aerobic activity.

The ET protocol included a continuous 2 h cycling session performed at 70–85% of the participants’ maximum heart rate (HR), immediately followed by a cycling bout at maximum HR until volitional exhaustion. The session concluded with a 5 min cool-down phase consisting of low-intensity cycling at 30–40% of HR and static stretching exercises.

The HIIT protocol consisted of two consecutive high-intensity segments:

1. 10 × 1 min bouts of cycling at 70% HR, each followed by 1 min rest periods.
2. 10 × 45 s running bouts performed at 80% to maximal HR, interspersed with 20 s passive recovery intervals.

The HIIT session also ended with a 5 min cool-down including jogging at 30–40% of HR and stretching.

All sessions were supervised, and heart rate was continuously monitored to ensure adherence to the target intensities.

The HIIT session was structured into two distinct phases based on intensity. The first phase involved moderate intensity, while the second phase, as instructed by the coaches, required maximum exertion. The protocols were designed to reflect realistic training practices used in elite wrestling preparation camps. They differed not only in exercise intensity but also in modality, duration, and work–rest structure. Therefore, the comparison should be interpreted as a comparison between two distinct training modalities rather than an isolated intensity-based contrast.

### 2.4. Anthropometric Measurements

All anthropometric assessments were conducted by the same researcher on the first day of the national team training camp, prior to breakfast. Body weight was measured using a calibrated electronic scale and recorded in kilograms. Stature was measured in meters using a wall-mounted stadiometer, with participants standing barefoot and in an upright posture.

### 2.5. Heart Rate Variability Measurements

HRV was assessed at multiple time points: daily for one week prior to each exercise protocol (baseline), on the morning of the exercise session (pre), during the exercise (training), and 24 h post-exercise (post 24 h). Morning measurements were taken in the supine position in bed immediately after waking, using 5 min recordings. During exercise, HRV data were continuously recorded throughout the entire duration of each training session, with no gaps in RR-interval acquisition, to compute average values.

Each participant was equipped with a Polar H10 chest strap heart rate monitor (Polar Electro Oy, Kempele, Finland), capable of sampling at 1000 Hz. The Polar H10 has been validated for accurate RR interval acquisition and HRV analysis at rest and during incremental exercise [23]. HRV data were recorded and exported via the Elite HRV mobile application (v5.5.8; Elite HRV, Inc., Asheville, NC, USA). Before data collection, participants were trained on how to use both the chest strap and the app.

Resting HRV measurements were obtained over a 5 min period each morning, immediately after waking and while lying in bed. For exercise measurements, the chest strap was securely positioned on the chest so as not to interfere with physical movement, and real-time monitoring was conducted. RR interval recordings were visually inspected within the Elite HRV platform. Abnormal beats and artefacts identified by the software’s automatic filtering algorithm were corrected through interpolation according to the manufacturer’s procedures. Signal quality was categorized automatically by the application as Good, Okay, or Poor; all retained recordings, at rest and during the TRAINING phase, were rated Good. The manufacturer does not disclose the specific artefact-detection and correction algorithm used [24]. HRV values obtained during exercise represent average values derived from the complete training session. No exercise segment was excluded unless signal loss prevented reliable RR interval acquisition; in the present dataset, no recordings or segments met this exclusion criterion, and complete TRAINING-phase data were available for all 13 participants under both protocols.

The HRV analysis included both time-domain (e.g., SDNN, RMSSD) and frequency-domain (e.g., LF/HF ratio) parameters. Mean HRV values were calculated for each time point (BASE, PRE, TRAINING, and POST 24 h), separately for the ET and HIIT protocols. In addition to these parameters, the lnRMSSD-derived HRV Score was computed from the natural logarithm of RMSSD (lnRMSSD), which the Elite HRV application expands onto a 0–100 scale using a proprietary, non-linear algorithm calibrated against its own database of recorded readings, rather than a fixed linear transformation. This composite score is automatically computed and exported by the application and has been used as an indicator of autonomic nervous system status in athlete monitoring [25,26]. Because the lnRMSSD-derived HRV Score is generated via a proprietary, non-linear, database-calibrated algorithm rather than a fixed transformation of RMSSD, raw RMSSD (together with SDNN) was treated as the primary time-domain HRV outcome in the present study; the lnRMSSD-derived HRV Score was treated as a secondary, exploratory outcome.

### 2.6. Statistical Analyses

HRV parameters—including SDNN, RMSSD, lnRMSSD-derived HRV Score, LF/HF ratio, and heart rate—were analyzed using a Linear Mixed Model to assess the main effects of Training Type (ET vs. HIIT), Timepoint (BASE, PRE, TRAINING, and POST 24 h), and their interaction (Timepoint × Training Type). Participant ID was included as a random effect with random intercepts to account for within-subject variability due to the repeated-measures design. The model was specified as HRV parameter~Training Type × Timepoint + (1 | Participant ID), fitted via restricted maximum likelihood (REML) using the GAMLj module in jamovi; degrees of freedom for fixed-effects tests were estimated using the Kenward–Roger approximation, and confidence intervals were constructed using the Wald method. Model assumption checks—residual normality (Shapiro–Wilk and Kolmogorov–Smirnov tests) and identification of extreme residuals by cluster—were conducted for each parameter within the GAMLj module; full diagnostic output is provided in File S1 (Supplementary Materials). No statistical outlier removal was performed; data inclusion was governed solely by the pre-specified signal-quality criterion described in Section 2.5.

Estimated Marginal Means (EMMs) were calculated for each level of the fixed factors, and Bonferroni-adjusted post hoc pairwise comparisons were conducted to explore significant differences across timepoints and between training types. Bonferroni correction was applied across all 28 pairwise Timepoint × Training Type comparisons per parameter to control the family-wise Type I error rate given the multiplicity of post hoc tests; we acknowledge that this conservative correction, combined with the small sample size, reduces power to detect true between-protocol differences, several of which showed large uncorrected effect sizes (see Section 3.1 and Section 3.4). Statistical significance was set at *p* < 0.05. Effect sizes were reported using partial eta squared (η^2^) for main and interaction effects, and Cohen’s d was calculated for relevant pairwise comparisons to interpret the magnitude of between-group differences at each timepoint (interpreted as: small ≥ 0.20, medium ≥ 0.50, large ≥ 0.80).

**Figure 2:**
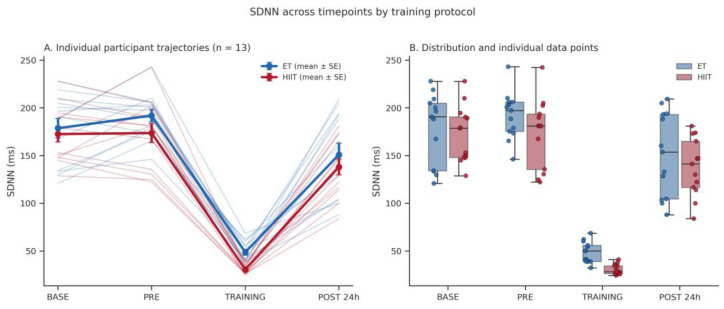
Change in SDNN Over Time by Training Type. (**A**) Individual participant trajectories (n = 13 per protocol) across four time points (BASE, PRE, TRAINING, POST 24 h), with protocol mean ± SE overlaid for ET and HIIT. (**B**) Distribution of SDNN (ms) at each time point by training type, shown as box plots with individual data points overlaid. Main effect of Timepoint: *p* < 0.001. Main effect of Training Type: *p* = 0.011. Post hoc between-protocol comparisons: not significant at any timepoint.

**Figure 3:**
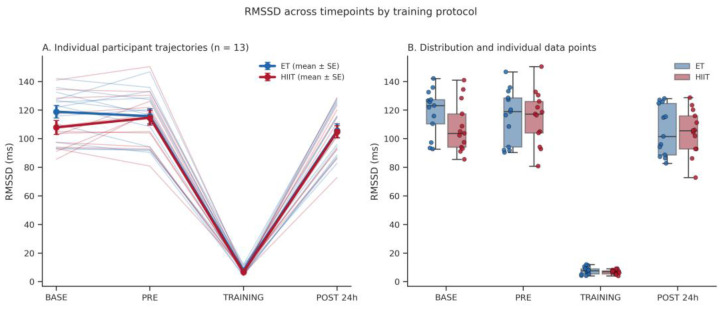
Change in RMSSD Over Time by Training Type. (**A**) Individual participant trajectories (n = 13 per protocol) across four time points (BASE, PRE, TRAINING, POST 24 h), with protocol mean ± SE overlaid for ET and HIIT. (**B**) Distribution of RMSSD (ms) at each time point by training type, shown as box plots with individual data points overlaid. Main effect of Timepoint: *p* < 0.001. Main effect of Training Type and Timepoint × Training Type interaction: not significant.

**Figure 4:**
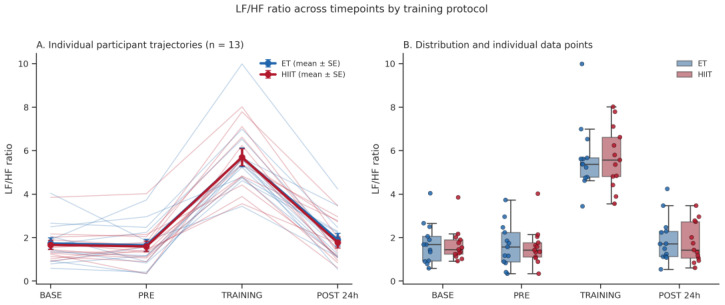
Change in LF/HF Ratio Over Time by Training Type. (**A**) Individual participant trajectories (n = 13 per protocol) across four time points (BASE, PRE, TRAINING, POST 24 h), with protocol mean ± SE overlaid for ET and HIIT. (**B**) Distribution of the LF/HF ratio at each time point by training type, shown as box plots with individual data points overlaid. Main effect of Timepoint: *p* < 0.001. Main effect of Training Type and Timepoint × Training Type interaction: not significant.

**Figure 5:**
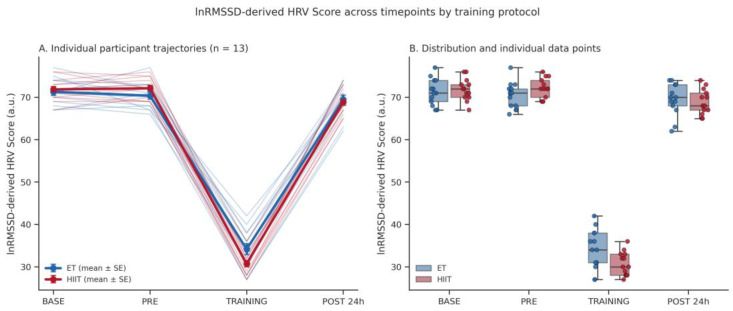
Change in lnRMSSD-derived HRV Score Over Time by Training Type. (**A**) Individual participant trajectories (n = 13 per protocol) across four time points (BASE, PRE, TRAINING, POST 24 h), with protocol mean ± SE overlaid for ET and HIIT. (**B**) Distribution of the lnRMSSD-derived HRV Score (a.u.) at each time point by training type, shown as box plots with individual data points overlaid. Main effect of Timepoint: *p* < 0.001. Timepoint × Training Type interaction: *p* = 0.008. Post hoc (TRAINING, HIIT vs. ET): *p* = 0.065, d = 0.79, not significant after Bonferroni correction.

**Figure 6:**
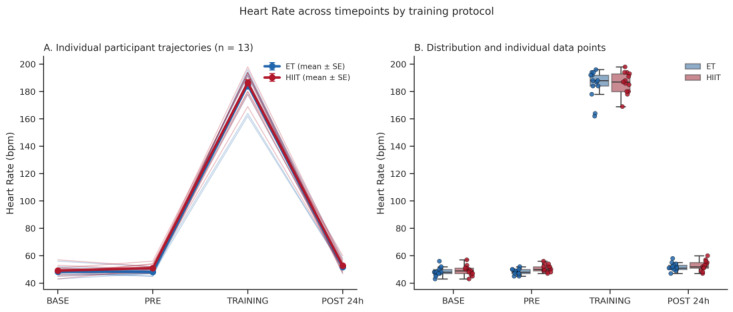
Change in Heart Rate Over Time by Training Type. (**A**) Individual participant trajectories (n = 13 per protocol) across four time points (BASE, PRE, TRAINING, POST 24 h), with protocol mean ± SE overlaid for ET and HIIT. (**B**) Distribution of heart rate (bpm) at each time point by training type, shown as box plots with individual data points overlaid. Main effect of Timepoint: *p* < 0.001. Main effect of Training Type and Timepoint × Training Type interaction: not significant.

All statistical analyses were conducted using jamovi (version 2.6.19.0), with linear mixed models fitted via the GAMLj module (version 3.6.5; R version 4.5). The authors additionally implemented an independent replication of the core model results in Python (v3.14.6; Python Software Foundation) using the statsmodels package (v0.14.6); the full replication script is provided in File S2 (Supplementary Materials). This Python analysis generated the individual-participant and distribution panels in Figure 2, Figure 3, Figure 4, Figure 5 and Figure 6; Claude (Anthropic) was used to assist in generating and troubleshooting this Python code under direct author supervision, and all code, outputs, and statistical interpretations were reviewed, run, and verified by the authors themselves. This replication reproduced jamovi’s fixed-effects F-tests (matching its Kenward–Roger-based results to within rounding error across all main and interaction effects for all five parameters) as well as its random-effects variance estimates.

## 3. Results

A total of 13 elite male Greco-Roman wrestlers participated in this within-subject, repeated measures design study. Each participant underwent both exercise conditions—ET and HIIT—with an adequate washout period between sessions to minimize potential carryover effects. The athletes had a mean age of 21.26 ± 1.34 years, mean body mass of 74.82 ± 8.01 kg, and mean height of 177.07 ± 6.56 cm. Since the study followed a within-subject, repeated measures design with a fixed protocol order, no between-group comparisons were required for baseline characteristics; HRV was instead compared within the same individuals across the two sequentially administered protocols. Estimated marginal means, between-condition mean differences, 95% confidence intervals, and effect sizes for all HRV and cardiovascular parameters are presented in Table 1. Model residuals showed no significant departure from normality for SDNN (Shapiro–Wilk W = 0.988, *p* = 0.463), RMSSD (W = 0.991, *p* = 0.750), the LF/HF ratio (W = 0.983, *p* = 0.195), and the lnRMSSD-derived HRV Score (W = 0.985, *p* = 0.274), with no extreme residuals identified for any of these four parameters; heart rate residuals departed significantly from normality (W = 0.912, *p* < 0.001) and are attributed to pronounced variance compression during the TRAINING phase (see Limitations). Full model diagnostic output is provided in File S1 (Supplementary Materials).

### 3.1. SDNN (Standard Deviation of NN Intervals)

The linear mixed model revealed a significant main effect of Timepoint on SDNN values (F(3, 84) = 156.475, *p* < 0.001, and η^2^ = 0.848), indicating substantial fluctuations across the measurement phases (Figure 2). Both training conditions (ET and HIIT) showed a pronounced decrease in SDNN during the TRAINING phase compared to BASE and PRE, followed by partial recovery at POST 24 h.

Additionally, a significant main effect of Training Type was found (F(1, 84) = 6.807, *p* = 0.011, and η^2^ = 0.075), suggesting that overall SDNN values differed between endurance and interval training modalities. However, the Timepoint × Training Type interaction was not statistically significant (F(3, 84) = 0.289, *p* = 0.833), indicating that the temporal response pattern was similar for both exercise types.

Post hoc comparisons did not reveal any statistically significant differences between the ET and HIIT groups at individual time points. The largest between-group difference occurred during the TRAINING phase (Cohen’s d = 1.43), a large effect size; however, this difference was not statistically significant after Bonferroni correction (*p* = 1.000), reflecting the wide confidence interval associated with the small sample.

### 3.2. RMSSD (Root Mean Square of Successive Differences)

The linear mixed model revealed a significant main effect of Timepoint on RMSSD values (F(3, 84) = 388.826, *p* < 0.001, and η^2^ = 0.933), indicating a substantial reduction in parasympathetic activity during the TRAINING phase in both exercise modalities (Figure 3). RMSSD decreased markedly during training compared to BASE and PRE, followed by partial recovery at POST 24 h.

However, the main effect of Training Type was not significant (F(1, 84) = 1.713, *p* = 0.194), nor was the Timepoint × Training interaction (F(3, 84) = 0.897, *p* = 0.446), suggesting that both groups exhibited similar temporal HRV responses.

Post hoc analysis showed no significant differences between ET and HIIT at any timepoint. The between-group effect size at the TRAINING phase was small (Cohen’s d = 0.36, ns).

### 3.3. LF/HF Ratio

A significant main effect of Timepoint was observed for the LF/HF ratio (F(3, 84) = 143.384, *p* < 0.001, and η^2^ = 0.837), indicating a significant shift in the LF/HF ratio during the TRAINING phase in both groups (Figure 4). This pattern is consistent with the typical cardiovascular response to acute physical stress; however, it should be noted that the validity of LF/HF ratio as an index of sympathovagal balance is limited during high-intensity exercise, as elevated breathing frequencies may encroach upon the LF band and confound frequency-domain interpretation [27,28].

However, no significant main effect of Training Type was found (F(1, 84) = 0.136, *p* = 0.713), and the Timepoint × Training interaction was also not significant (F(3, 84) = 0.027, *p* = 0.994).

These results indicate that ET and HIIT produced comparable shifts in the LF/HF ratio over time, though the autonomic interpretation of this index during exercise should be treated with caution. The effect size during TRAINING was negligible (Cohen’s d = 0.00) and not statistically significant.

### 3.4. lnRMSSD-Derived HRV Score

The linear mixed model revealed a robust main effect of Timepoint on HRV (F(3, 84) = 1260.976, *p* < 0.001, η^2^ = 0.978), indicating substantial temporal fluctuations across conditions (Figure 5). A significant Timepoint × Training Type interaction was also observed (F(3, 84) = 4.164, *p* = 0.008, η^2^ = 0.129), indicating that the magnitude of the ET–HIIT difference changed across timepoints; specifically, the shift in this difference from BASE to TRAINING was significant (interaction contrast estimate = −3.92, SE = 1.52, *p* = 0.012). The main effect of Training Type was not significant (F(1, 84) = 0.561, *p* = 0.456). However, Bonferroni-corrected post hoc comparisons found no statistically significant difference between ET and HIIT specifically at the TRAINING timepoint (Diff = 3.39, *p* = 0.065), despite a large effect size (Cohen’s d = 0.79)—a pattern consistent with a true difference that the present sample (n = 13) was underpowered to confirm at the corrected significance threshold.

### 3.5. Heart Rate

Linear mixed model analysis demonstrated a highly significant main effect of Timepoint on heart rate (F(3, 84) = 4973.270, *p* < 0.001, η^2^ = 0.994), indicating a substantial increase in HR during the TRAINING phase, followed by a return toward baseline values at POST 24 h (Figure 6). In contrast, neither the main effect of Training Type (F(1, 84) = 2.376, *p* = 0.127) nor the Timepoint × Training interaction (F(3, 84) = 0.234, *p* = 0.872) reached statistical significance, suggesting that both ET and HIIT protocols elicited similar heart rate responses over time. The between-group effect size during TRAINING was small (Cohen’s d = 0.23, not significant), implying only minimal differences in cardiovascular load during the exercise sessions.

## 4. Discussion

The present study investigated acute HRV responses to two different training modalities—cycling-based ET and HIIT—in elite Greco-Roman wrestlers. The primary aim was to evaluate the autonomic responses during exercise and to assess the extent of recovery 24 h post-exercise by analyzing multiple HRV indices. Given the importance of HRV as a non-invasive marker of ANS function, this study also sought to determine the potential utility of HRV for monitoring training load and recovery status in elite athletes. While the pattern of pronounced acute HRV suppression during exercise, with partial recovery by 24 h, is consistent with the general exercise physiology literature, the present study contributes to knowledge in several specific ways. First, this is, to our knowledge, the first study to examine acute HRV responses to these two training modalities in elite Greco-Roman wrestlers, a population that has been largely overlooked in HRV research despite its distinct physiological and competitive demands. Second, HRV was monitored continuously across four ecologically valid time points, including during actual training sessions rather than restricted to pre- and post-exercise resting measurements, providing a more complete picture of autonomic dynamics. Third, the data were collected within a real national team preparatory camp, rather than a controlled laboratory setting, enhancing the applied relevance of the findings for practitioners working with elite combat sports athletes. As protocol order was fixed rather than randomized, the following interpretations should be read as pertaining to these two specific field-based conditioning sessions rather than as an isolated test of training intensity.

Our findings revealed that baseline HRV values (BASE and PRE) were comparable between the two training conditions, indicating a similar autonomic state prior to exercise regardless of training type. However, both ET and HIIT elicited significant reductions in HRV indices, particularly SDNN, RMSSD, and lnRMSSD-derived HRV Score during the TRAINING phase, accompanied by an increase in the LF/HF ratio and heart rate—the former interpreted here only as a descriptive frequency-domain change rather than a direct index of sympathovagal balance, given the known confound of elevated respiratory frequency during high-intensity exercise [27]. These changes are broadly consistent with a shift toward sympathetic dominance and parasympathetic withdrawal, which is consistent with the physiological stress response observed during acute physical exertion. This phenomenon has been well-documented in the literature, with acute exercise known to decrease HRV through mechanisms including increased heart rate, respiratory frequency, and blood pressure, all of which amplify cardiovascular and metabolic demand [28,29,30]. Such autonomic adjustments are indicative of the body’s effort to maintain homeostasis under physical load and are mediated primarily through the sympathetic branch of the ANS [31].

Because raw RMSSD—the primary time-domain outcome—showed no significant Timepoint × Training Type interaction, the following pattern observed in the secondary, proprietary HRV Score should be interpreted as exploratory. Despite similar overall trends, between-group comparisons revealed a numerically greater suppression of the lnRMSSD-derived HRV Score during the training session in the HIIT condition compared to ET, although this difference did not reach statistical significance after Bonferroni correction. This suggests that although both training methods impose substantial physiological stress, the specific HIIT protocol used in this investigation may have elicited a somewhat greater suppression of the lnRMSSD-derived HRV Score than the specific ET protocol examined, a pattern the present sample was not powered to confirm statistically. Nevertheless, because the protocols differed simultaneously in intensity, duration, exercise modality, and work–rest structure, the observed differences should not be interpreted as being attributable to exercise intensity alone.

This numerically greater suppression of the lnRMSSD-derived HRV Score in the HIIT group, although not statistically significant after correction, may be attributable to the combined influence of higher exercise intensity, the intermittent work–rest structure, the inclusion of running as a second modality, and the overall shorter session duration compared to ET. Because the two protocols differed across multiple dimensions simultaneously, this pattern cannot be attributed to exercise intensity alone. This tentative pattern is nonetheless consistent with studies demonstrating that high-intensity interval protocols can induce pronounced acute autonomic responses [29,32] and underscores the importance of considering protocol structure when interpreting HRV data in field-based settings.

These findings may have potential practical relevance for coaches and sport scientists, as they illustrate the physiological demands imposed by HIIT relative to a longer ET session. Whether this pattern supports HIIT’s inclusion as a time-efficient alternative in high-performance training programs is a potential application that was not directly tested in the present study and would require further research examining training outcomes directly. Importantly, these findings contribute to the broader discussion of whether HRV monitoring may have utility for tracking acute exercise stress in elite populations, although the present study did not directly evaluate its use for real-time decision-making or recovery prescription. Neither heart rate nor the lnRMSSD-derived HRV Score differed significantly between protocols during the training phase after correction (*p* = 0.127 and *p* = 0.065, respectively). It is also worth noting that the Timepoint × Training Type interaction was significant for the lnRMSSD-derived HRV Score (*p* = 0.008) but not for raw RMSSD (F(3, 84) = 0.897, *p* = 0.446), even though the two metrics are mathematically related. This divergence is consistent with the fact that the Elite HRV application does not apply a simple fixed multiplier to lnRMSSD but rather a proprietary, non-linear, database-calibrated scaling [26], which can amplify or attenuate between-condition differences relative to the raw RMSSD signal. These findings support the growing proposition that HRV-based metrics, rather than or in addition to heart rate, may serve as real-time indices of exercise intensity and internal load in elite sport settings [28].

Overall, the acute decrease in HRV during both exercise conditions highlights the responsiveness of the ANS to physical load, while the comparable suppression observed during HIIT and ET reflects the combined demands of each protocol rather than exercise intensity alone. While both modalities led to partial recovery at the 24 h post-exercise mark, the sensitivity of HRV to these acute changes suggests its potential value as a monitoring tool, though its application to individualized training design was not directly tested in the present study.

Although changes in HRV during exercise are primarily driven by the imposed training load and the athlete’s fitness level, post-exercise HRV fluctuations—especially those measured at rest—are modulated by a broader range of physiological and psychological factors, including sleep quality, nutritional status, emotional stress, and circadian influences [33]. In the present study, given that all participants were residing in the same training camp under standardized living conditions with uniform training loads and schedules, it is reasonable to assume that variability in extraneous factors influencing HRV (e.g., sleep, diet, and environmental stressors) was reduced, though not eliminated, relative to a less controlled setting. This partial standardization should be weighed against the measurement limitations noted in the Limitations section when interpreting the 24 h post-exercise HRV assessments.

Although no statistically significant between-protocol differences were observed at the 24 h post-exercise timepoint, SDNN and RMSSD values tended to remain numerically lower following HIIT compared to ET. This pattern may suggest a slower autonomic recovery trend after high-intensity exercise; however, these observations should be interpreted with caution. This finding highlights the sensitivity of these time-domain indices to post-exercise parasympathetic activity and supports their use as markers of recovery. Elevated post-exercise SDNN values are typically associated with increased vagal tone and reduced sympathetic dominance, both of which are recognized as indicators of favorable recovery status and cardiovascular fitness [15,33]. Therefore, the numerically higher SDNN values observed after ET may reflect a tendency toward greater autonomic recovery, although no statistically significant differences were detected between protocols at the 24 h timepoint.

Although between-protocol differences at 24 h did not reach statistical significance, the observed numerical trend is consistent with prior studies reporting reduced HRV values in the hours and days following strenuous training sessions [34,35]. These patterns suggest that training intensity may play a role in shaping post-exercise autonomic recovery trajectories, a possibility that warrants investigation with extended monitoring windows. Taken together, our results are consistent with the broader literature suggesting a potential role for SDNN and RMSSD as tools for evaluating short-term recovery; however, the present study did not directly test their use for guiding training decisions, and this application remains to be demonstrated in future research.

Finally, regardless of the fluctuations in HRV observed during and after exercise sessions, the athletes in this study consistently demonstrated high resting HRV and SDNN values throughout the daily monitoring period. Sustained elevations in daily SDNN are widely considered a marker of optimal autonomic balance and physiological readiness, indicating that an athlete is well-prepared for subsequent training or competition [36,37]. Elevated resting SDNN values commonly observed in elite endurance and high-performance athletes reflect a highly adaptable ANS, capable of efficiently responding to and recovering from physical stressors such as intensive training loads.

More specifically, high SDNN values suggest enhanced flexibility in both the sympathetic and parasympathetic branches of the ANS, contributing to robust cardiovascular health and superior aerobic fitness. This autonomic efficiency facilitates rapid post-exercise recovery by promoting effective parasympathetic reactivation (the “rest-and-digest” response), which accelerates physiological repair processes. Accordingly, the elevated resting HRV and SDNN values observed in the athletes of this study likely reflect their elite performance status and may serve as reliable biomarkers of training readiness and overall physiological resilience in the lead-up to competition.

Conversely, this study has several limitations that should be considered. First, the small and homogeneous sample limited to 13 elite male Greco-Roman wrestlers restricts the generalizability of the findings to other populations, such as female athletes or competitors from different sports. Second, while the camp environment ensured similar training and living conditions, factors known to influence HRV, including sleep quality, caffeine intake, hydration status, nutritional intake, and psychological stress, were not objectively measured. Therefore, their potential influence on autonomic responses cannot be excluded. Third, post-exercise recovery was only assessed at a single time point (24 h), which may not fully capture the autonomic recovery process, particularly after high-intensity training. Fourth, the fixed protocol sequence (ET preceding HIIT) represents a design limitation, as potential cumulative fatigue, training adaptations, or other time-dependent effects cannot be fully excluded despite the 15-day interval between sessions. Because the protocol order was determined by the national team coaching staff, changes occurring during the training camp may have contributed to the observed differences between protocols. Additionally, because TRAINING-phase HRV was averaged across each entire session, and the ET and HIIT sessions differed substantially in total duration, the resulting averages do not represent comparable time windows and may be differentially influenced by within-session pacing or fatigue effects. A sensitivity analysis restricted to standardized, equal-duration segments of each session was not feasible with the available data and is recommended for future studies directly comparing protocols of different duration. Fifth, the exercise protocols consisted of cycling- and running-based conditioning rather than wrestling-specific technical or tactical training. While these modalities are authentically used in elite wrestling preparation, the acute HRV responses observed here may not fully reflect the autonomic demands of mat-based wrestling training, which involves different neuromuscular, biomechanical, and psychological stressors. Future studies should examine HRV responses during and after wrestling-specific training formats to complement the present findings. Furthermore, the use of the LF/HF ratio as an index of sympathovagal balance during the TRAINING phase is subject to methodological limitations. During high-intensity exercise, respiratory frequency may rise into the LF band, rendering standard frequency-domain interpretation unreliable [27]. Accordingly, LF/HF values reported during exercise should be interpreted as descriptive indicators of frequency-domain change rather than direct measures of autonomic balance. Although the Polar H10 chest strap has been validated for RR-interval acquisition during incremental exercise [23], software-level validation of the Elite HRV application’s artefact-correction output against an independent criterion has, to our knowledge, been demonstrated only at rest (ICC = 0.938–0.998, [38]; r = 0.92 with a systematic negative bias [24]) and has not been established for high-intensity continuous exercise; this is acknowledged as a limitation of the present TRAINING-phase HRV measurements. Additionally, within-cell variance differed substantially across timepoints for several HRV parameters (e.g., RMSSD SD ≈ 2 ms during TRAINING versus 17–19 ms at BASE/PRE/POST 24 h), reflecting the well-documented compression of time-domain HRV during high-intensity exercise; because the mixed model assumes homogeneous residual variance, the TRAINING-phase confidence intervals in Table 1 should be interpreted with this in mind. Consistent with this variance compression, heart rate model residuals showed a significant departure from normality (Shapiro–Wilk W = 0.912, *p* < 0.001), whereas residuals for the other four parameters did not (all *p* ≥ 0.195), with no extreme residuals identified for any parameter. This HR-specific deviation should be considered when interpreting HR-related inferential statistics, although the corresponding effect estimates and confidence intervals in Table 1 remain informative as descriptive summaries. Lastly, the absence of additional physiological or biochemical markers limits interpretation of recovery dynamics. Future studies should combine HRV with complementary recovery measures and longer monitoring periods.

## 5. Conclusions

This study examined the acute autonomic responses and subsequent recovery following two distinct training modalities—cycling-based ET and HIIT—in elite Greco-Roman wrestlers preparing for international competition. By employing continuous HRV monitoring across key time points (baseline, during exercise, and 24 h post-exercise), the study provided detailed insights into how distinct training modalities differing in intensity, structure, and duration impact ANS dynamics. Both protocols reduced HRV during exercise and showed partial recovery within 24 h. Greater suppression was observed during the HIIT protocol for the secondary, proprietary lnRMSSD-derived HRV Score, a pattern not observed in the primary raw RMSSD outcome; because the protocols differed in duration, modality, and structure, and because this pattern rests on a single exploratory, non-transparent metric, it should be interpreted with particular caution.

The consistently high baseline HRV values observed throughout the study are consistent with the well-developed autonomic regulation expected in elite athletes; whether this observation translates into a demonstrated usefulness of HRV monitoring in high-performance settings was not directly tested here and remains a potential application. The fixed order of the protocols and the small sample size further warrant cautious interpretation.

In conclusion, the present findings should be regarded as exploratory, given the small sample size and the use of two training protocols that differed simultaneously in intensity, duration, modality, and structure. Within these constraints, HRV monitoring may provide complementary information regarding acute training responses, autonomic stress, and short-term recovery in elite wrestlers. Future studies should include larger, wrestling-specific cohorts, randomized and protocol-matched designs, and longer recovery monitoring periods to confirm and extend the present findings.

## 6. Practical Applications

The present findings support the sensitivity of HRV metrics to acute exercise stress. HRV monitoring, particularly during and immediately after sessions, may serve as a practical, non-invasive tool for quantifying acute physiological stress. These autonomic markers may complement other training-load indicators already used by coaches and practitioners, although the present study did not directly evaluate their use for real-time decision-making or load prescription.

Additionally, the present findings suggest that individual HRV responses may be useful to consider when interpreting acute physiological responses to different training sessions. Although both HIIT and ET demonstrated partial recovery within 24 h post-exercise, continued HRV monitoring may provide further information regarding recovery patterns and inter-individual variability following exercise.

Finally, the findings suggest that HRV may have potential utility within broader athlete monitoring frameworks. To further enhance athlete monitoring, future applications should incorporate long-term HRV surveillance in conjunction with other physiological or perceptual metrics.

## Figures and Tables

**Table 1:** Estimated marginal means, between-condition mean differences, 95% confidence intervals, and effect sizes for HRV and cardiovascular parameters across timepoints and training conditions.

Parameter	Time-Point	ET M (SD)	HIIT M (SD)	Diff ± ME (ET − HIIT)	*p* (Bonf.)	d
SDNN (ms)	BASE	178.7 (37.2)	172.6 (29.5)	6.14 ± 21.08	1.000	0.23
	PRE	191.9 (24.5)	173.7 (36.2)	18.29 ± 21.08	0.334	0.65
	TRAINING	48.7 (11.1)	30.8 (5.4)	17.93 ± 21.08	0.359	1.43
	POST 24 h	150.9 (44.0)	138.1 (30.2)	12.77 ± 21.08	0.907	0.23
RMSSD (ms)	BASE	118.9 (16.1)	107.9 (17.5)	10.99 ± 10.52	0.152	0.59
	PRE	115.8 (18.9)	114.7 (19.0)	1.05 ± 10.52	1.000	0.07
	TRAINING	7.8 (2.6)	6.8 (1.6)	0.98 ± 10.52	1.000	0.36
	POST 24 h	105.7 (17.5)	104.8 (15.8)	0.84 ± 10.52	1.000	0.03
LF/HF ratio	BASE	1.7 (0.9)	1.7 (0.8)	0.07 ± 0.66	1.000	0.06
	PRE	1.6 (1.0)	1.6 (0.9)	0.05 ± 0.66	1.000	0.06
	TRAINING	5.7 (1.6)	5.7 (1.4)	0.00 ± 0.66	1.000	0.00
	POST 24 h	1.9 (1.0)	1.8 (0.9)	0.13 ± 0.66	1.000	0.12
lnRMSSD (a.u.)	BASE	71.3 (3.1)	71.8 (2.6)	−0.54 ± 2.15	1.000	−0.11
	PRE	70.4 (3.1)	72.2 (2.4)	−1.77 ± 2.15	0.403	−0.46
	TRAINING	34.1 (4.8)	30.8 (2.8)	3.39 ± 2.15	0.065	0.79
	POST 24 h	69.5 (3.8)	68.9 (3.0)	0.54 ± 2.15	1.000	0.13
Heart Rate (bpm)	BASE	48.4 (3.3)	49.1 (3.6)	−0.69 ± 3.82	1.000	−0.25
	PRE	48.1 (2.1)	50.9 (2.7)	−2.77 ± 3.82	0.598	−1.08
	TRAINING	184.8 (10.9)	186.4 (8.0)	−1.54 ± 3.82	1.000	−0.23
	POST 24 h	51.8 (2.8)	52.7 (3.7)	−0.92 ± 3.82	1.000	−0.33

Note. M (SD) = raw within-cell mean (standard deviation), n = 13 per cell. Diff ± ME = mixed-model estimated mean difference (ET − HIIT) ± margin of error of the 95% confidence interval (CI = Diff ± ME), from the linear mixed model (Training × Timepoint, random intercept for participant). *p* values are Wald-test based and Bonferroni-corrected across all pairwise Timepoint × Training comparisons for that parameter (28 comparisons per parameter). Cohen’s d was calculated from paired within-subject differences (small ≥ 0.20, medium ≥ 0.50, large ≥ 0.80). Omnibus main and interaction effects for each parameter (F, df, *p*, η^2^) are reported in the corresponding Section 3.1, Section 3.2, Section 3.3, Section 3.4 and Section 3.5.

## Data Availability

Data and methods of analysis are available to qualified researchers upon request.

## Supplementary Materials

The following supporting information can be downloaded at: https://www.mdpi.com/article/10.3390/sports14070299/s1; File S1: Full jamovi/GAMLj statistical output (model specification, fixed- and random-effects estimates, post hoc pairwise comparisons, estimated marginal means, and residual diagnostics) for all five HRV parameters; File S2: Python script (HRV_mixed_model_python_replication.py) used to independently replicate the mixed models (variance components, ICC, and Shapiro–Wilk normality tests are produced when the script is run). References [39,40,41,42,43] are cited in File S1.
