# Supplementary material for: Investigating the Acute Effect of Different Training Protocols on Heart Rate Variability
Source: Sports (Basel). 2026 Jul 13;14(7):299. doi: 10.3390/sports14070299 (PMC13416595; doi:10.3390/sports14070299)
Supplement: Supplementary file 1 [file sports-14-00299-s001.zip › S1- Statistics .pdf]

# Results

## Mixed Model

Model Info

| Info | | |
|---|---|---|
| Model Type | Mixed Model | Linear Mixed model for continuous y |
| Model | lmer | HRV ~ 1 + Timepoint + Training + Timepoint:Training + ( 1 \| ID ) |
| Distribution | Gaussian | Normal distribution of residuals |
| Direction | y | Dependend variable scores |
| Optimizer | bobyqa | |
| DF method | Kenward-Roger | |
| Sample size | 104 | |
| Converged | yes | |
| Y transform | none | |
| C.I. method | Wald | |

**[3]**

### Model Results

Model Fit

| Type | $R^2$ | df | LRT $X^2$ | p |
|---|---|---|---|---|
| Conditional | 0.974 | 8 | 366.917 | <.001 |
| Marginal | 0.963 | 7 | 366.917 | <.001 |

**[4]**

Fixed Effects Omnibus Tests

| | F | df | df (res) | p |
|---|---|---|---|---|
| Timepoint | 1260.976 | 3 | 84.0 | <.001 |
| Training | 0.561 | 1 | 84.0 | .456 |
| Timepoint ✻ Training | 4.164 | 3 | 84.0 | .008 |

Parameter Estimates (Fixed coefficients)

| | | | | 95% Confidence Intervals | | | | |
|---|---|---|---|---|---|---|---|---|
| Names | Effect | Estimate | SE | Lower | Upper | df | t | p |
| (Intercept) | (Intercept) | 61.125 | 0.564 | 60.01 | 62.245 | 12.0 | 108.375 | <.001 |
| Timepoint1 | 2 - 1 | -0.308 | 0.762 | -1.82 | 1.206 | 84.0 | -0.404 | .687 |
| Timepoint2 | 3 - 1 | -39.115 | 0.762 | -40.63 | -37.602 | 84.0 | -51.320 | <.001 |
| Timepoint3 | 4 - 1 | -2.385 | 0.762 | -3.90 | -0.871 | 84.0 | -3.129 | .002 |
| Training1 | HIIT - ET | -0.404 | 0.539 | -1.47 | 0.666 | 84.0 | -0.749 | .456 |
| Timepoint1 ✻ Training1 | (2 - 1) ✻ (HIIT - ET) | 1.231 | 1.524 | -1.80 | 4.257 | 84.0 | 0.807 | .422 |
| Timepoint2 ✻ Training1 | (3 - 1) ✻ (HIIT - ET) | -3.923 | 1.524 | -6.95 | -0.896 | 84.0 | -2.574 | .012 |
| Timepoint3 ✻ Training1 | (4 - 1) ✻ (HIIT - ET) | -1.077 | 1.524 | -4.10 | 1.950 | 84.0 | -0.706 | .482 |

**[5]**

Random Components

| Groups | Name | Variance | SD | ICC |
|---|---|---|---|---|
| ID | (Intercept) | 3.19 | 1.79 | 0.297 |
| Residual | | 7.55 | 2.75 | |

Note. Number of Obs: 104 , Number of groups: ID 13

## Post Hoc Tests

Post Hoc comparison: Timepoint ✻ Training

| Comparison | | | | | | | | | |
|---|---|---|---|---|---|---|---|---|---|
| Timepoint | Training | vs | Timepoint | Training | Difference | SE | t | df | $p_{bonferroni}$ |
| 1 | ET | - | 1 | HIIT | -0.538 | 1.08 | -0.500 | 84.0 | 1.000 |
| 1 | ET | - | 2 | ET | 0.923 | 1.08 | 0.856 | 84.0 | 1.000 |
| 1 | ET | - | 2 | HIIT | -0.846 | 1.08 | -0.785 | 84.0 | 1.000 |
| 1 | ET | - | 3 | ET | 37.154 | 1.08 | 34.469 | 84.0 | <.001 |
| 1 | ET | - | 3 | HIIT | 40.538 | 1.08 | 37.609 | 84.0 | <.001 |
| 1 | ET | - | 4 | ET | 1.846 | 1.08 | 1.713 | 84.0 | 1.000 |
| 1 | ET | - | 4 | HIIT | 2.385 | 1.08 | 2.212 | 84.0 | .831 |
| 1 | HIIT | - | 2 | ET | 1.462 | 1.08 | 1.356 | 84.0 | 1.000 |
| 1 | HIIT | - | 2 | HIIT | -0.308 | 1.08 | -0.285 | 84.0 | 1.000 |
| 1 | HIIT | - | 3 | ET | 37.692 | 1.08 | 34.968 | 84.0 | <.001 |
| 1 | HIIT | - | 3 | HIIT | 41.077 | 1.08 | 38.108 | 84.0 | <.001 |
| 1 | HIIT | - | 4 | ET | 2.385 | 1.08 | 2.212 | 84.0 | .831 |
| 1 | HIIT | - | 4 | HIIT | 2.923 | 1.08 | 2.712 | 84.0 | .227 |
| 2 | ET | - | 2 | HIIT | -1.769 | 1.08 | -1.641 | 84.0 | 1.000 |
| 2 | ET | - | 3 | ET | 36.231 | 1.08 | 33.612 | 84.0 | <.001 |
| 2 | ET | - | 3 | HIIT | 39.615 | 1.08 | 36.752 | 84.0 | <.001 |
| 2 | ET | - | 4 | ET | 0.923 | 1.08 | 0.856 | 84.0 | 1.000 |
| 2 | ET | - | 4 | HIIT | 1.462 | 1.08 | 1.356 | 84.0 | 1.000 |
| 2 | HIIT | - | 3 | ET | 38.000 | 1.08 | 35.254 | 84.0 | <.001 |
| 2 | HIIT | - | 3 | HIIT | 41.385 | 1.08 | 38.394 | 84.0 | <.001 |
| 2 | HIIT | - | 4 | ET | 2.692 | 1.08 | 2.498 | 84.0 | .405 |
| 2 | HIIT | - | 4 | HIIT | 3.231 | 1.08 | 2.997 | 84.0 | .100 |
| 3 | ET | - | 3 | HIIT | 3.385 | 1.08 | 3.140 | 84.0 | .065 |
| 3 | ET | - | 4 | ET | -35.308 | 1.08 | -32.756 | 84.0 | <.001 |
| 3 | ET | - | 4 | HIIT | -34.769 | 1.08 | -32.256 | 84.0 | <.001 |
| 3 | HIIT | - | 4 | ET | -38.692 | 1.08 | -35.896 | 84.0 | <.001 |
| 3 | HIIT | - | 4 | HIIT | -38.154 | 1.08 | -35.396 | 84.0 | <.001 |
| 4 | ET | - | 4 | HIIT | 0.538 | 1.08 | 0.500 | 84.0 | 1.000 |

## Estimated Marginal Means

Estimate Marginal Means - Timepoint ✻ Training

| | | | | | 95% Confidence Intervals | |
|---|---|---|---|---|---|---|
| Timepoint | Training | Mean | SE | df | Lower | Upper |
| 1 | ET | 71.3 | 0.909 | 59.3 | 69.5 | 73.1 |
| 1 | HIIT | 71.8 | 0.909 | 59.3 | 70.0 | 73.7 |
| 2 | ET | 70.4 | 0.909 | 59.3 | 68.6 | 72.2 |
| 2 | HIIT | 72.2 | 0.909 | 59.3 | 70.3 | 74.0 |
| 3 | ET | 34.2 | 0.909 | 59.3 | 32.3 | 36.0 |
| 3 | HIIT | 30.8 | 0.909 | 59.3 | 29.0 | 32.6 |
| 4 | ET | 69.5 | 0.909 | 59.3 | 67.6 | 71.3 |
| 4 | HIIT | 68.9 | 0.909 | 59.3 | 67.1 | 70.7 |

Estimate Marginal Means - Timepoint

| | | | | 95% Confidence Intervals | |
|---|---|---|---|---|---|
| Timepoint | Mean | SE | df | Lower | Upper |
| 1 | 71.6 | 0.732 | 31.9 | 70.1 | 73.1 |
| 2 | 71.3 | 0.732 | 31.9 | 69.8 | 72.8 |
| 3 | 32.5 | 0.732 | 31.9 | 31.0 | 34.0 |
| 4 | 69.2 | 0.732 | 31.9 | 67.7 | 70.7 |

Estimate Marginal Means - Training

| | | | | 95% Confidence Intervals | |
|---|---|---|---|---|---|
| Training | Mean | SE | df | Lower | Upper |
| ET | 61.3 | 0.625 | 18.0 | 60.0 | 62.6 |
| HIIT | 60.9 | 0.625 | 18.0 | 59.6 | 62.2 |

## Results Plots

**Plot: HRV ~ Timepoint ✻ Training**

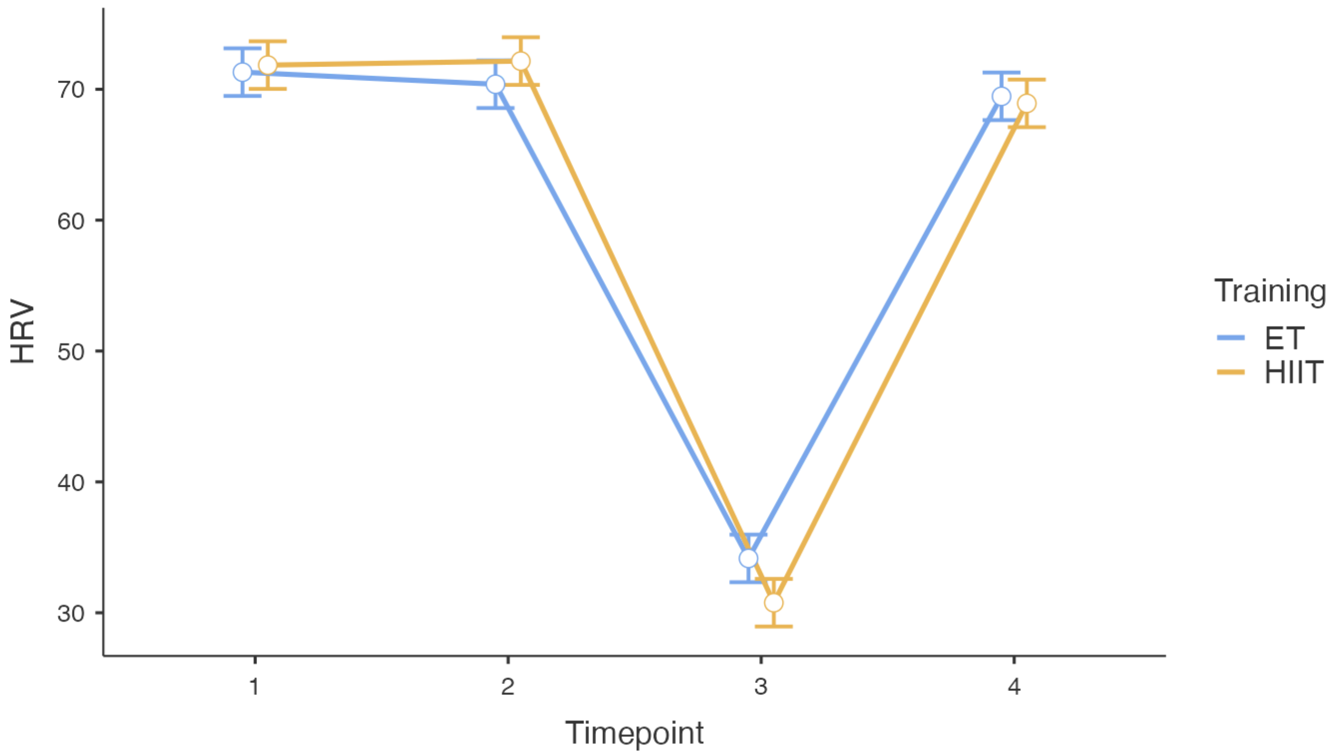


## Assumption Checks

Test for Normality of residuals

| Test | Statistics | p |
|---|---|---|
| Kolmogorov-Smirnov | 0.0571 | .887 |
| Shapiro-Wilk | 0.9846 | .274 |

**Q-Q Plot**

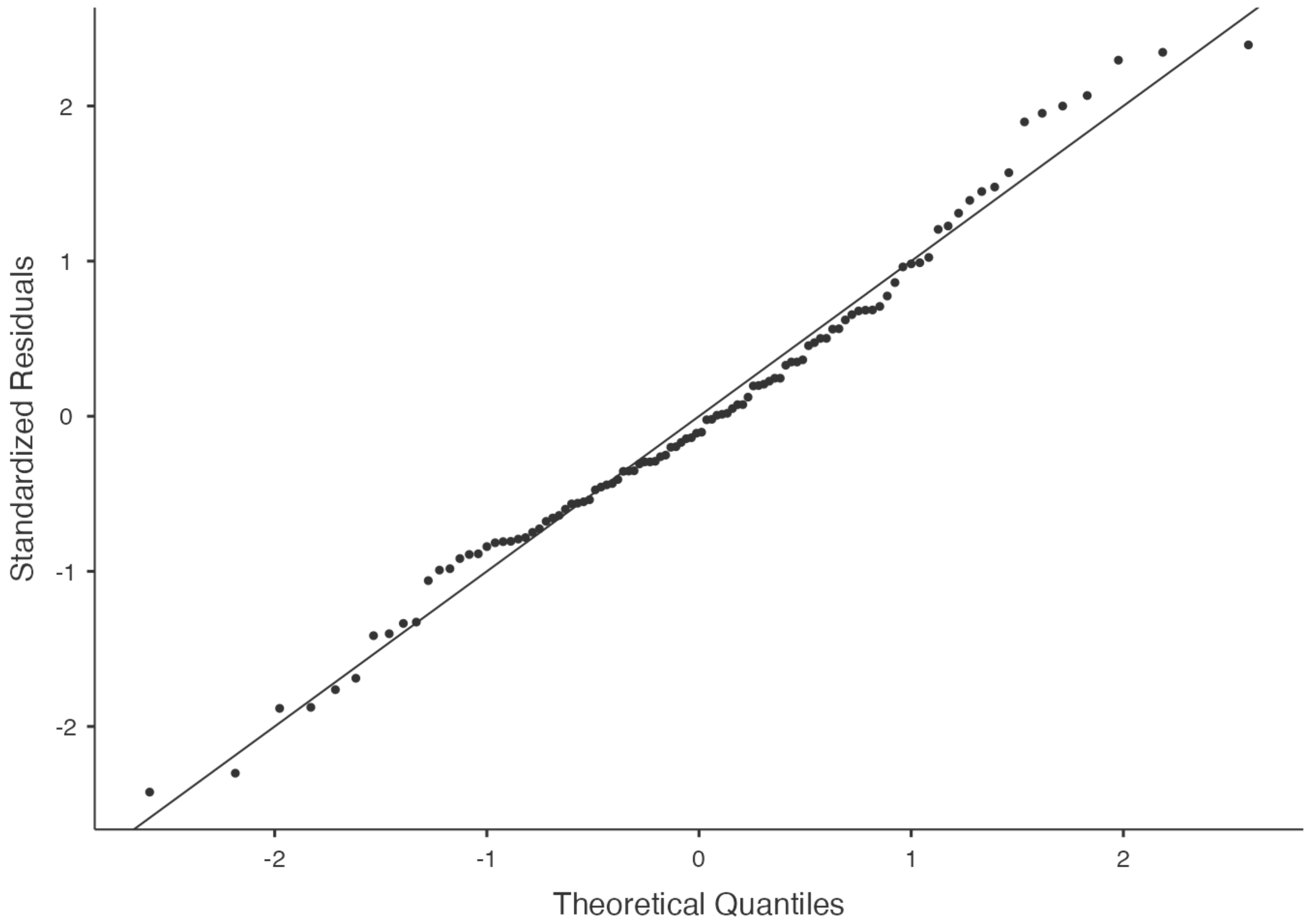


## Mixed Model

Model Info

| Info | | |
|---|---|---|
| Model Type | Mixed Model | Linear Mixed model for continuous y |
| Model | lmer | SDNN ~ 1 + Timepoint + Training + Timepoint:Training + ( 1 \| ID ) |
| Distribution | Gaussian | Normal distribution of residuals |
| Direction | y | Dependend variable scores |
| Optimizer | bobyqa | |
| DF method | Kenward-Roger | |
| Sample size | 104 | |
| Converged | yes | |
| Y transform | none | |
| C.I. method | Wald | |

**[3]**

### Model Results

Model Fit

| Type | $R^2$ | df | LRT $X^2$ | p |
|---|---|---|---|---|
| Conditional | 0.830 | 8 | 176.101 | <.001 |
| Marginal | 0.790 | 7 | 176.101 | <.001 |

**[4]**

Fixed Effects Omnibus Tests

| | F | df | df (res) | p |
|---|---|---|---|---|
| Timepoint | 156.475 | 3 | 84.0 | <.001 |
| Training | 6.807 | 1 | 84.0 | .011 |
| Timepoint ✻ Training | 0.289 | 3 | 84.0 | .833 |

Parameter Estimates (Fixed coefficients)

| | | | | 95% Confidence Intervals | | | | |
|---|---|---|---|---|---|---|---|---|
| Names | Effect | Estimate | SE | Lower | Upper | df | t | p |
| (Intercept) | (Intercept) | 135.67 | 4.48 | 126.78 | 144.56 | 12.0 | 30.300 | <.001 |
| Timepoint1 | 2 - 1 | 7.13 | 7.47 | -7.70 | 21.97 | 84.0 | 0.955 | .342 |
| Timepoint2 | 3 - 1 | -135.95 | 7.47 | -150.79 | -121.12 | 84.0 | -18.193 | <.001 |
| Timepoint3 | 4 - 1 | -31.17 | 7.47 | -46.00 | -16.33 | 84.0 | -4.171 | <.001 |
| Training1 | HIIT - ET | -13.79 | 5.28 | -24.28 | -3.29 | 84.0 | -2.609 | .011 |
| Timepoint1 ✻ Training1 | (2 - 1) ✻ (HIIT - ET) | -12.15 | 14.95 | -41.83 | 17.52 | 84.0 | -0.813 | .418 |
| Timepoint2 ✻ Training1 | (3 - 1) ✻ (HIIT - ET) | -11.79 | 14.95 | -41.47 | 17.88 | 84.0 | -0.789 | .432 |
| Timepoint3 ✻ Training1 | (4 - 1) ✻ (HIIT - ET) | -6.63 | 14.95 | -36.31 | 23.04 | 84.0 | -0.444 | .658 |

**[5]**

Random Components

| Groups | Name | Variance | SD | ICC |
|---|---|---|---|---|
| ID | (Intercept) | 170 | 13.0 | 0.190 |
| Residual | | 726 | 26.9 | |

Note. Number of Obs: 104 , Number of groups: ID 13

## Post Hoc Tests

Post Hoc comparison: Timepoint ✻ Training

| Comparison | | | | | | | | | |
|---|---|---|---|---|---|---|---|---|---|
| Timepoint | Training | vs | Timepoint | Training | Difference | SE | t | df | $p_{bonferroni}$ |
| 1 | ET | - | 1 | HIIT | 6.14 | 10.6 | 0.581 | 84.0 | 1.000 |
| 1 | ET | - | 2 | ET | -13.21 | 10.6 | -1.250 | 84.0 | 1.000 |
| 1 | ET | - | 2 | HIIT | 5.08 | 10.6 | 0.481 | 84.0 | 1.000 |
| 1 | ET | - | 3 | ET | 130.06 | 10.6 | 12.307 | 84.0 | <.001 |
| 1 | ET | - | 3 | HIIT | 147.99 | 10.6 | 14.004 | 84.0 | <.001 |
| 1 | ET | - | 4 | ET | 27.85 | 10.6 | 2.635 | 84.0 | .280 |
| 1 | ET | - | 4 | HIIT | 40.62 | 10.6 | 3.844 | 84.0 | .007 |
| 1 | HIIT | - | 2 | ET | -19.35 | 10.6 | -1.831 | 84.0 | 1.000 |
| 1 | HIIT | - | 2 | HIIT | -1.06 | 10.6 | -0.100 | 84.0 | 1.000 |
| 1 | HIIT | - | 3 | ET | 123.92 | 10.6 | 11.726 | 84.0 | <.001 |
| 1 | HIIT | - | 3 | HIIT | 141.85 | 10.6 | 13.423 | 84.0 | <.001 |
| 1 | HIIT | - | 4 | ET | 21.71 | 10.6 | 2.054 | 84.0 | 1.000 |
| 1 | HIIT | - | 4 | HIIT | 34.48 | 10.6 | 3.263 | 84.0 | .045 |
| 2 | ET | - | 2 | HIIT | 18.29 | 10.6 | 1.731 | 84.0 | 1.000 |
| 2 | ET | - | 3 | ET | 143.27 | 10.6 | 13.557 | 84.0 | <.001 |
| 2 | ET | - | 3 | HIIT | 161.20 | 10.6 | 15.254 | 84.0 | <.001 |
| 2 | ET | - | 4 | ET | 41.06 | 10.6 | 3.885 | 84.0 | .006 |
| 2 | ET | - | 4 | HIIT | 53.83 | 10.6 | 5.094 | 84.0 | <.001 |
| 2 | HIIT | - | 3 | ET | 124.97 | 10.6 | 11.826 | 84.0 | <.001 |
| 2 | HIIT | - | 3 | HIIT | 142.91 | 10.6 | 13.523 | 84.0 | <.001 |
| 2 | HIIT | - | 4 | ET | 22.77 | 10.6 | 2.154 | 84.0 | .954 |
| 2 | HIIT | - | 4 | HIIT | 35.54 | 10.6 | 3.363 | 84.0 | .033 |
| 3 | ET | - | 3 | HIIT | 17.93 | 10.6 | 1.697 | 84.0 | 1.000 |
| 3 | ET | - | 4 | ET | -102.21 | 10.6 | -9.671 | 84.0 | <.001 |
| 3 | ET | - | 4 | HIIT | -89.43 | 10.6 | -8.463 | 84.0 | <.001 |
| 3 | HIIT | - | 4 | ET | -120.14 | 10.6 | -11.368 | 84.0 | <.001 |
| 3 | HIIT | - | 4 | HIIT | -107.37 | 10.6 | -10.160 | 84.0 | <.001 |
| 4 | ET | - | 4 | HIIT | 12.77 | 10.6 | 1.209 | 84.0 | 1.000 |

## Estimated Marginal Means

Estimate Marginal Means - Timepoint ✻ Training

| | | | | | 95% Confidence Intervals | |
|---|---|---|---|---|---|---|
| Timepoint | Training | Mean | SE | df | Lower | Upper |
| 1 | ET | 178.7 | 8.30 | 76.7 | 162.2 | 195.3 |
| 1 | HIIT | 172.6 | 8.30 | 76.7 | 156.1 | 189.1 |
| 2 | ET | 192.0 | 8.30 | 76.7 | 175.4 | 208.5 |
| 2 | HIIT | 173.7 | 8.30 | 76.7 | 157.1 | 190.2 |
| 3 | ET | 48.7 | 8.30 | 76.7 | 32.2 | 65.2 |
| 3 | HIIT | 30.7 | 8.30 | 76.7 | 14.2 | 47.3 |
| 4 | ET | 150.9 | 8.30 | 76.7 | 134.4 | 167.4 |
| 4 | HIIT | 138.1 | 8.30 | 76.7 | 121.6 | 154.6 |

Estimate Marginal Means - Timepoint

| | | | | 95% Confidence Intervals | |
|---|---|---|---|---|---|
| Timepoint | Mean | SE | df | Lower | Upper |
| 1 | 175.7 | 6.40 | 43.4 | 162.8 | 188.6 |
| 2 | 182.8 | 6.40 | 43.4 | 169.9 | 195.7 |
| 3 | 39.7 | 6.40 | 43.4 | 26.8 | 52.6 |
| 4 | 144.5 | 6.40 | 43.4 | 131.6 | 157.4 |

Estimate Marginal Means - Training

| | | | | 95% Confidence Intervals | |
|---|---|---|---|---|---|
| Training | Mean | SE | df | Lower | Upper |
| ET | 143 | 5.20 | 21.4 | 132 | 153 |
| HIIT | 129 | 5.20 | 21.4 | 118 | 140 |

## Results Plots

**Plot: SDNN ~ Timepoint ✻ Training**

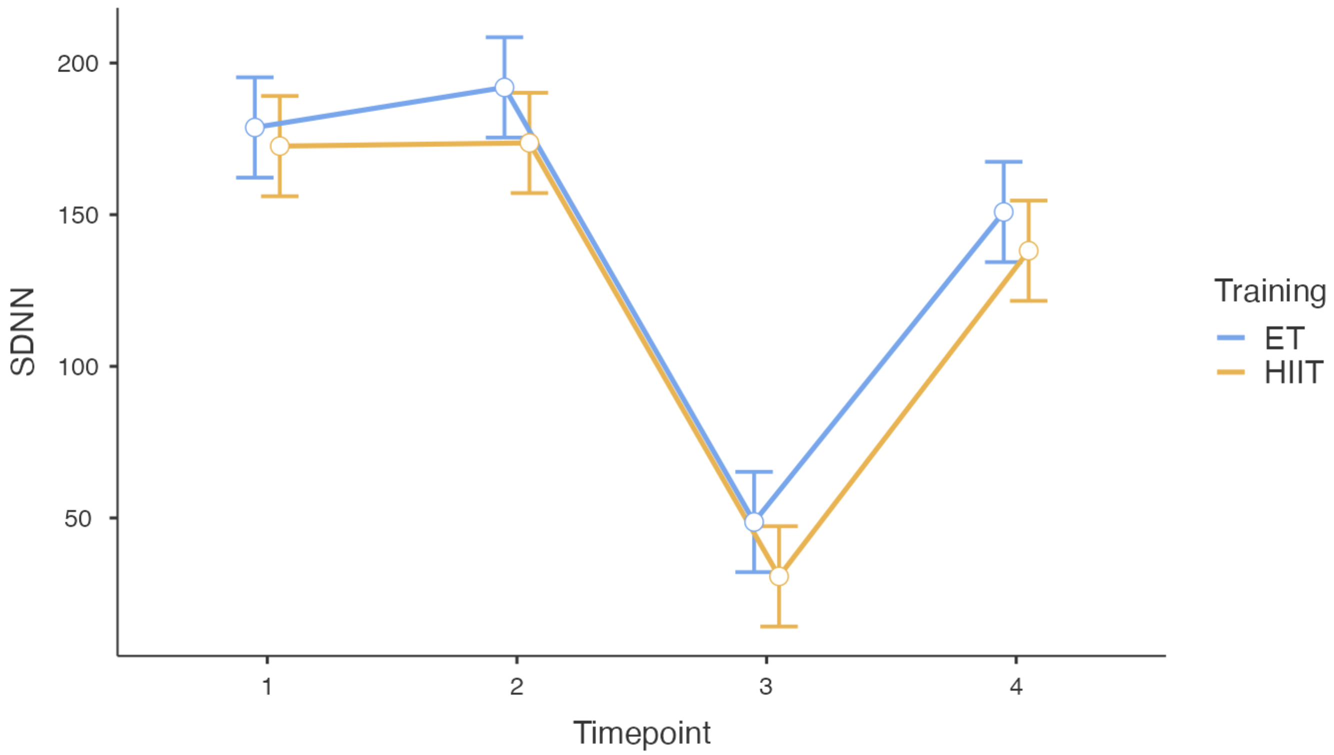


## Assumption Checks

Test for Normality of residuals

| Test | Statistics | p |
|---|---|---|
| Kolmogorov-Smirnov | 0.0738 | .623 |
| Shapiro-Wilk | 0.9878 | .463 |

### Q-Q Plot

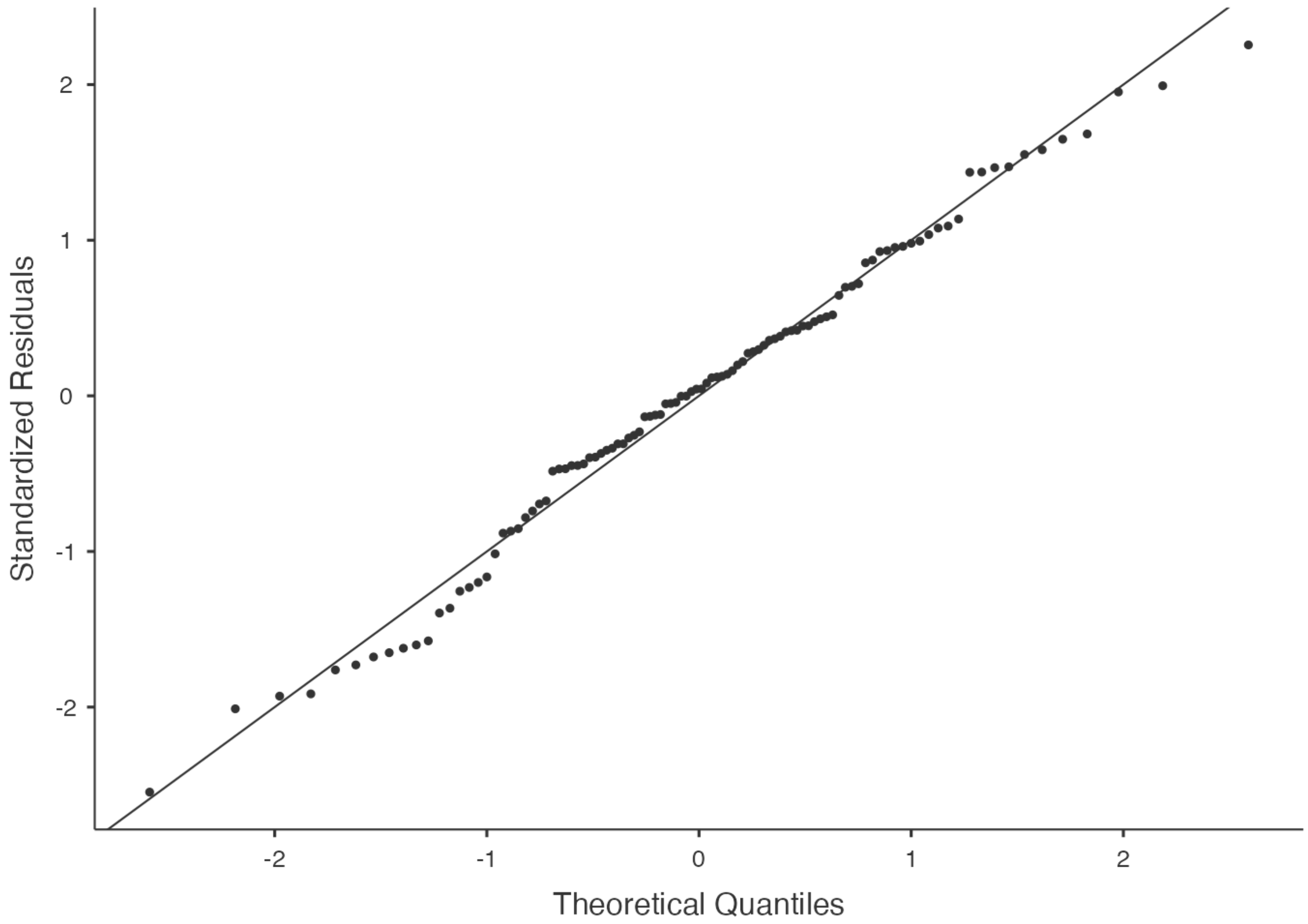


# Mixed Model

Model Info

| Info | | |
|---|---|---|
| Model Type | Mixed Model | Linear Mixed model for continuous y |
| Model | lmer | RMSSD ~ 1 + Timepoint + Training + Timepoint:Training + ( 1 \| ID ) |
| Distribution | Gaussian | Normal distribution of residuals |
| Direction | y | Dependend variable scores |
| Optimizer | bobyqa | |
| DF method | Kenward-Roger | |
| Sample size | 104 | |
| Converged | yes | |
| Y transform | none | |
| C.I. method | Wald | |

**[3]**

## Model Results

Model Fit

| Type | $R^2$ | df | LRT $X^2$ | p |
|---|---|---|---|---|
| Conditional | 0.921 | 8 | 255.561 | <.001 |
| Marginal | 0.900 | 7 | 255.561 | <.001 |

**[4]**

Fixed Effects Omnibus Tests

| | F | df | df (res) | p |
|---|---|---|---|---|
| Timepoint | 388.826 | 3 | 84.0 | <.001 |
| Training | 1.713 | 1 | 84.0 | .194 |
| Timepoint ✻ Training | 0.897 | 3 | 84.0 | .446 |

Parameter Estimates (Fixed coefficients)

| | | | | 95% Confidence Intervals | | | | |
|---|---|---|---|---|---|---|---|---|
| Names | Effect | Estimate | SE | Lower | Upper | df | t | p |
| (Intercept) | (Intercept) | 85.28 | 2.34 | 80.63 | 89.924 | 12.0 | 36.428 | <.001 |
| Timepoint1 | 2 - 1 | 1.84 | 3.74 | -5.59 | 9.273 | 84.0 | 0.492 | .624 |
| Timepoint2 | 3 - 1 | -106.11 | 3.74 | -113.54 | -98.682 | 84.0 | -28.352 | <.001 |
| Timepoint3 | 4 - 1 | -8.16 | 3.74 | -15.59 | -0.727 | 84.0 | -2.180 | .032 |
| Training1 | HIIT - ET | -3.46 | 2.65 | -8.72 | 1.790 | 84.0 | -1.309 | .194 |
| Timepoint1 ✻ Training1 | (2 - 1) ✻ (HIIT - ET) | 9.93 | 7.49 | -4.93 | 24.795 | 84.0 | 1.327 | .188 |
| Timepoint2 ✻ Training1 | (3 - 1) ✻ (HIIT - ET) | 10.01 | 7.49 | -4.86 | 24.869 | 84.0 | 1.337 | .185 |
| Timepoint3 ✻ Training1 | (4 - 1) ✻ (HIIT - ET) | 10.14 | 7.49 | -4.72 | 25.005 | 84.0 | 1.355 | .179 |

**[5]**

Random Components

| Groups | Name | Variance | SD | ICC |
|---|---|---|---|---|
| ID | (Intercept) | 48.5 | 6.96 | 0.210 |
| Residual | | 182.1 | 13.49 | |

Note. Number of Obs: 104 , Number of groups: ID 13

## Post Hoc Tests

Post Hoc comparison: Timepoint ✻ Training

| Comparison | | | | | | | | | |
|---|---|---|---|---|---|---|---|---|---|
| Timepoint | Training | vs | Timepoint | Training | Difference | SE | t | df | $p_{bonferroni}$ |
| 1 | ET | - | 1 | HIIT | 10.985 | 5.29 | 2.075 | 84.0 | 1.000 |
| 1 | ET | - | 2 | ET | 3.125 | 5.29 | 0.590 | 84.0 | 1.000 |
| 1 | ET | - | 2 | HIIT | 4.177 | 5.29 | 0.789 | 84.0 | 1.000 |
| 1 | ET | - | 3 | ET | 111.116 | 5.29 | 20.993 | 84.0 | <.001 |
| 1 | ET | - | 3 | HIIT | 112.095 | 5.29 | 21.178 | 84.0 | <.001 |
| 1 | ET | - | 4 | ET | 13.230 | 5.29 | 2.500 | 84.0 | .403 |
| 1 | ET | - | 4 | HIIT | 14.072 | 5.29 | 2.659 | 84.0 | .263 |
| 1 | HIIT | - | 2 | ET | -7.860 | 5.29 | -1.485 | 84.0 | 1.000 |
| 1 | HIIT | - | 2 | HIIT | -6.808 | 5.29 | -1.286 | 84.0 | 1.000 |
| 1 | HIIT | - | 3 | ET | 100.132 | 5.29 | 18.918 | 84.0 | <.001 |
| 1 | HIIT | - | 3 | HIIT | 101.110 | 5.29 | 19.103 | 84.0 | <.001 |
| 1 | HIIT | - | 4 | ET | 2.245 | 5.29 | 0.424 | 84.0 | 1.000 |
| 1 | HIIT | - | 4 | HIIT | 3.087 | 5.29 | 0.583 | 84.0 | 1.000 |
| 2 | ET | - | 2 | HIIT | 1.052 | 5.29 | 0.199 | 84.0 | 1.000 |
| 2 | ET | - | 3 | ET | 107.992 | 5.29 | 20.403 | 84.0 | <.001 |
| 2 | ET | - | 3 | HIIT | 108.970 | 5.29 | 20.588 | 84.0 | <.001 |
| 2 | ET | - | 4 | ET | 10.105 | 5.29 | 1.909 | 84.0 | 1.000 |
| 2 | ET | - | 4 | HIIT | 10.947 | 5.29 | 2.068 | 84.0 | 1.000 |
| 2 | HIIT | - | 3 | ET | 106.939 | 5.29 | 20.204 | 84.0 | <.001 |
| 2 | HIIT | - | 3 | HIIT | 107.918 | 5.29 | 20.389 | 84.0 | <.001 |
| 2 | HIIT | - | 4 | ET | 9.053 | 5.29 | 1.710 | 84.0 | 1.000 |
| 2 | HIIT | - | 4 | HIIT | 9.895 | 5.29 | 1.869 | 84.0 | 1.000 |
| 3 | ET | - | 3 | HIIT | 0.978 | 5.29 | 0.185 | 84.0 | 1.000 |
| 3 | ET | - | 4 | ET | -97.886 | 5.29 | -18.494 | 84.0 | <.001 |
| 3 | ET | - | 4 | HIIT | -97.045 | 5.29 | -18.335 | 84.0 | <.001 |
| 3 | HIIT | - | 4 | ET | -98.865 | 5.29 | -18.679 | 84.0 | <.001 |
| 3 | HIIT | - | 4 | HIIT | -98.023 | 5.29 | -18.520 | 84.0 | <.001 |
| 4 | ET | - | 4 | HIIT | 0.842 | 5.29 | 0.159 | 84.0 | 1.000 |

## Estimated Marginal Means

Estimate Marginal Means - Timepoint ✻ Training

| | | | | | 95% Confidence Intervals | |
|---|---|---|---|---|---|---|
| Timepoint | Training | Mean | SE | df | Lower | Upper |
| 1 | ET | 118.88 | 4.21 | 73.3 | 110.482 | 127.3 |
| 1 | HIIT | 107.89 | 4.21 | 73.3 | 99.498 | 116.3 |
| 2 | ET | 115.75 | 4.21 | 73.3 | 107.358 | 124.1 |
| 2 | HIIT | 114.70 | 4.21 | 73.3 | 106.306 | 123.1 |
| 3 | ET | 7.76 | 4.21 | 73.3 | -0.634 | 16.2 |
| 3 | HIIT | 6.78 | 4.21 | 73.3 | -1.612 | 15.2 |
| 4 | ET | 105.65 | 4.21 | 73.3 | 97.252 | 114.0 |
| 4 | HIIT | 104.80 | 4.21 | 73.3 | 96.411 | 113.2 |

Estimate Marginal Means - Timepoint

| | | | | 95% Confidence Intervals | |
|---|---|---|---|---|---|
| Timepoint | Mean | SE | df | Lower | Upper |
| 1 | 113.38 | 3.28 | 40.7 | 106.765 | 120.0 |
| 2 | 115.22 | 3.28 | 40.7 | 108.607 | 121.8 |
| 3 | 7.27 | 3.28 | 40.7 | 0.652 | 13.9 |
| 4 | 105.22 | 3.28 | 40.7 | 98.607 | 111.8 |

Estimate Marginal Means - Training

| | | | | 95% Confidence Intervals | |
|---|---|---|---|---|---|
| Training | Mean | SE | df | Lower | Upper |
| ET | 87.0 | 2.69 | 20.6 | 81.4 | 92.6 |
| HIIT | 83.5 | 2.69 | 20.6 | 77.9 | 89.1 |

## Results Plots

**Plot: RMSSD ~ Timepoint ✻ Training**

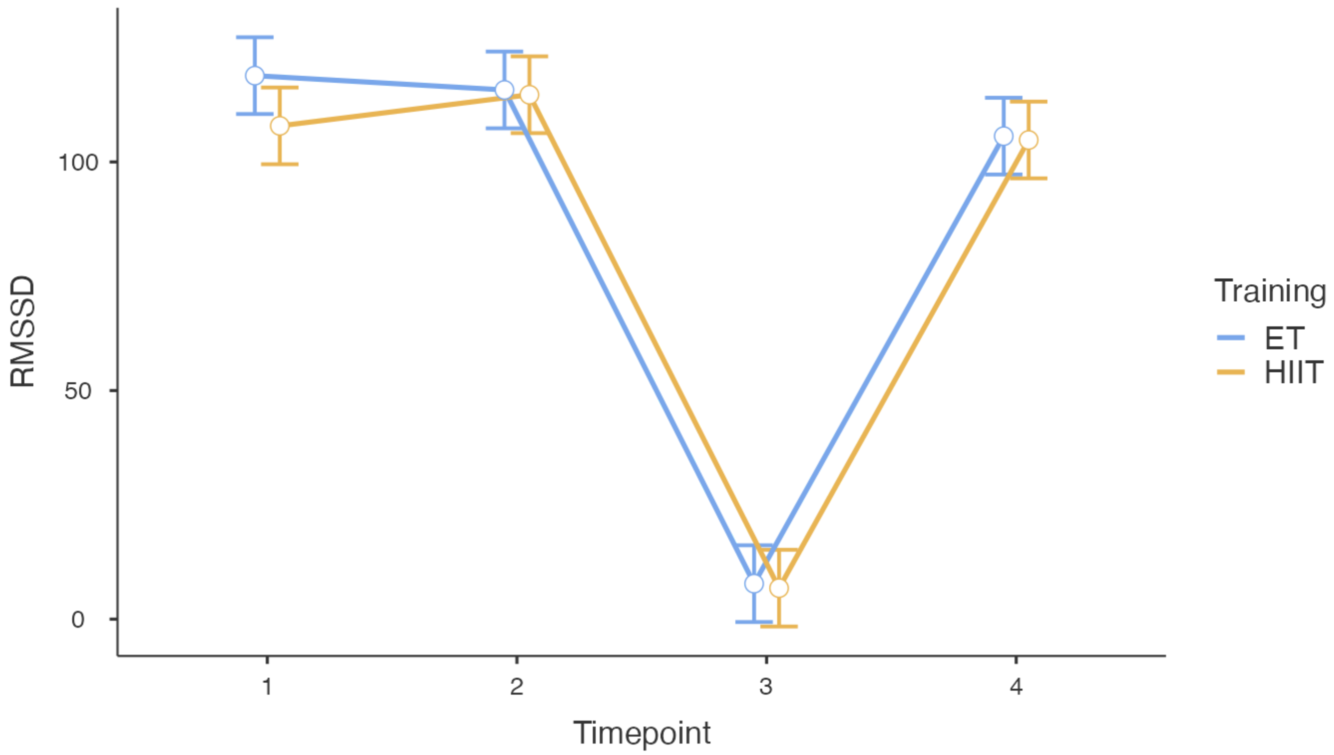


## Assumption Checks

Test for Normality of residuals

| Test | Statistics | p |
|---|---|---|
| Kolmogorov-Smirnov | 0.0471 | .975 |
| Shapiro-Wilk | 0.9914 | .750 |

### Q-Q Plot

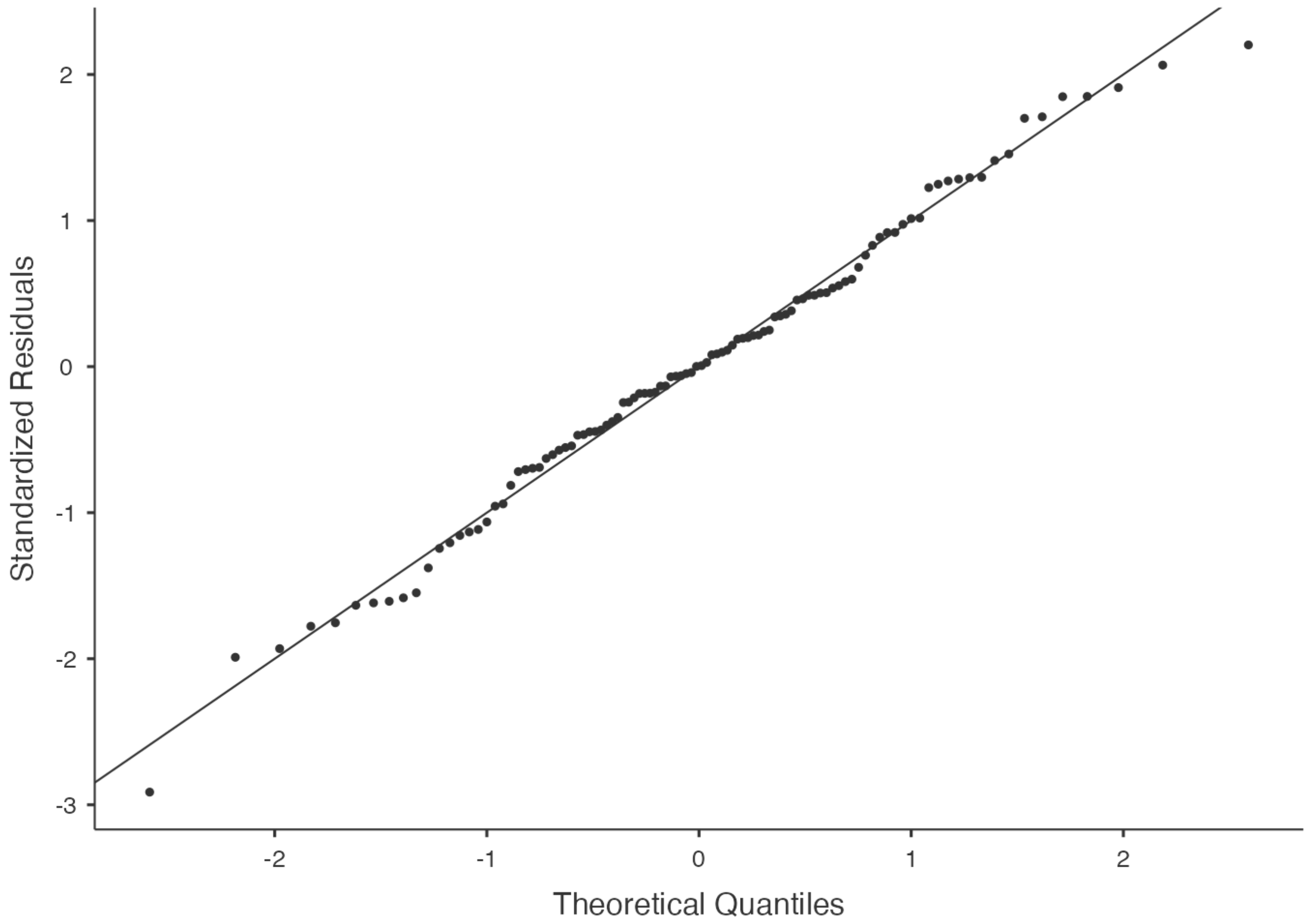


## Mixed Model

Model Info

| Info | | |
|---|---|---|
| Model Type | Mixed Model | Linear Mixed model for continuous y |
| Model | lmer | `LF/HF` ~ 1 + Timepoint + Training + Timepoint:Training + ( 1 \| ID ) |
| Distribution | Gaussian | Normal distribution of residuals |
| Direction | y | Dependend variable scores |
| Optimizer | bobyqa | |
| DF method | Kenward-Roger | |
| Sample size | 104 | |
| Converged | yes | |
| Y transform | none | |
| C.I. method | Wald | |

**[3]**

### Model Results

Model Fit

| Type | $R^2$ | df | LRT $X^2$ | p |
|---|---|---|---|---|
| Conditional | 0.829 | 8 | 164.915 | <.001 |
| Marginal | 0.714 | 7 | 164.939 | <.001 |

**[4]**

Fixed Effects Omnibus Tests

| | F | df | df (res) | p |
|---|---|---|---|---|
| Timepoint | 143.3843 | 3 | 84.0 | <.001 |
| Training | 0.1363 | 1 | 84.0 | .713 |
| Timepoint ✻ Training | 0.0266 | 3 | 84.0 | .994 |

Parameter Estimates (Fixed coefficients)

| | | | | 95% Confidence Intervals | | | | |
|---|---|---|---|---|---|---|---|---|
| Names | Effect | Estimate | SE | Lower | Upper | df | t | p |
| (Intercept) | (Intercept) | 2.7116 | 0.210 | 2.294 | 3.129 | 12.0 | 12.8963 | <.001 |
| Timepoint1 | 2 - 1 | -0.0792 | 0.235 | -0.546 | 0.387 | 84.0 | -0.3371 | .737 |
| Timepoint2 | 3 - 1 | 3.9965 | 0.235 | 3.530 | 4.463 | 84.0 | 17.0048 | <.001 |
| Timepoint3 | 4 - 1 | 0.1415 | 0.235 | -0.325 | 0.608 | 84.0 | 0.6022 | .549 |
| Training1 | HIIT - ET | -0.0613 | 0.166 | -0.391 | 0.269 | 84.0 | -0.3691 | .713 |
| Timepoint1 ✻ Training1 | (2 - 1) ✻ (HIIT - ET) | 0.0169 | 0.470 | -0.916 | 0.950 | 84.0 | 0.0360 | .971 |
| Timepoint2 ✻ Training1 | (3 - 1) ✻ (HIIT - ET) | 0.0700 | 0.470 | -0.863 | 1.003 | 84.0 | 0.1489 | .882 |
| Timepoint3 ✻ Training1 | (4 - 1) ✻ (HIIT - ET) | -0.0615 | 0.470 | -0.995 | 0.872 | 84.0 | -0.1309 | .896 |

**[5]**

Random Components

| Groups | Name | Variance | SD | ICC |
|---|---|---|---|---|
| ID | (Intercept) | 0.485 | 0.696 | 0.403 |
| Residual | | 0.718 | 0.847 | |

Note. Number of Obs: 104 , Number of groups: ID 13

## Post Hoc Tests

Post Hoc comparison: Timepoint ✻ Training

| Comparison | | | | | | | | | |
|---|---|---|---|---|---|---|---|---|---|
| Timepoint | Training | vs | Timepoint | Training | Difference | SE | t | df | $p_{bonferroni}$ |
| 1 | ET | - | 1 | HIIT | 0.06769 | 0.332 | 0.20366 | 84.0 | 1.000 |
| 1 | ET | - | 2 | ET | 0.08769 | 0.332 | 0.26384 | 84.0 | 1.000 |
| 1 | ET | - | 2 | HIIT | 0.13846 | 0.332 | 0.41658 | 84.0 | 1.000 |
| 1 | ET | - | 3 | ET | -3.96154 | 0.332 | -11.91889 | 84.0 | <.001 |
| 1 | ET | - | 3 | HIIT | -3.96385 | 0.332 | -11.92583 | 84.0 | <.001 |
| 1 | ET | - | 4 | ET | -0.17231 | 0.332 | -0.51841 | 84.0 | 1.000 |
| 1 | ET | - | 4 | HIIT | -0.04308 | 0.332 | -0.12960 | 84.0 | 1.000 |
| 1 | HIIT | - | 2 | ET | 0.02000 | 0.332 | 0.06017 | 84.0 | 1.000 |
| 1 | HIIT | - | 2 | HIIT | 0.07077 | 0.332 | 0.21292 | 84.0 | 1.000 |
| 1 | HIIT | - | 3 | ET | -4.02923 | 0.332 | -12.12255 | 84.0 | <.001 |
| 1 | HIIT | - | 3 | HIIT | -4.03154 | 0.332 | -12.12949 | 84.0 | <.001 |
| 1 | HIIT | - | 4 | ET | -0.24000 | 0.332 | -0.72208 | 84.0 | 1.000 |
| 1 | HIIT | - | 4 | HIIT | -0.11077 | 0.332 | -0.33327 | 84.0 | 1.000 |
| 2 | ET | - | 2 | HIIT | 0.05077 | 0.332 | 0.15275 | 84.0 | 1.000 |
| 2 | ET | - | 3 | ET | -4.04923 | 0.332 | -12.18272 | 84.0 | <.001 |
| 2 | ET | - | 3 | HIIT | -4.05154 | 0.332 | -12.18967 | 84.0 | <.001 |
| 2 | ET | - | 4 | ET | -0.26000 | 0.332 | -0.78225 | 84.0 | 1.000 |
| 2 | ET | - | 4 | HIIT | -0.13077 | 0.332 | -0.39344 | 84.0 | 1.000 |
| 2 | HIIT | - | 3 | ET | -4.10000 | 0.332 | -12.33547 | 84.0 | <.001 |
| 2 | HIIT | - | 3 | HIIT | -4.10231 | 0.332 | -12.34241 | 84.0 | <.001 |
| 2 | HIIT | - | 4 | ET | -0.31077 | 0.332 | -0.93500 | 84.0 | 1.000 |
| 2 | HIIT | - | 4 | HIIT | -0.18154 | 0.332 | -0.54619 | 84.0 | 1.000 |
| 3 | ET | - | 3 | HIIT | -0.00231 | 0.332 | -0.00694 | 84.0 | 1.000 |
| 3 | ET | - | 4 | ET | 3.78923 | 0.332 | 11.40047 | 84.0 | <.001 |
| 3 | ET | - | 4 | HIIT | 3.91846 | 0.332 | 11.78928 | 84.0 | <.001 |
| 3 | HIIT | - | 4 | ET | 3.79154 | 0.332 | 11.40742 | 84.0 | <.001 |
| 3 | HIIT | - | 4 | HIIT | 3.92077 | 0.332 | 11.79623 | 84.0 | <.001 |
| 4 | ET | - | 4 | HIIT | 0.12923 | 0.332 | 0.38881 | 84.0 | 1.000 |

## Estimated Marginal Means

Estimate Marginal Means - Timepoint ✻ Training

| | | | | | 95% Confidence Intervals | |
|---|---|---|---|---|---|---|
| Timepoint | Training | Mean | SE | df | Lower | Upper |
| 1 | ET | 1.73 | 0.304 | 44.9 | 1.118 | 2.34 |
| 1 | HIIT | 1.66 | 0.304 | 44.9 | 1.050 | 2.28 |
| 2 | ET | 1.64 | 0.304 | 44.9 | 1.030 | 2.26 |
| 2 | HIIT | 1.59 | 0.304 | 44.9 | 0.980 | 2.21 |
| 3 | ET | 5.69 | 0.304 | 44.9 | 5.080 | 6.31 |
| 3 | HIIT | 5.69 | 0.304 | 44.9 | 5.082 | 6.31 |
| 4 | ET | 1.90 | 0.304 | 44.9 | 1.290 | 2.52 |
| 4 | HIIT | 1.77 | 0.304 | 44.9 | 1.161 | 2.39 |

Estimate Marginal Means - Timepoint

| | | | | 95% Confidence Intervals | |
|---|---|---|---|---|---|
| Timepoint | Mean | SE | df | Lower | Upper |
| 1 | 1.70 | 0.255 | 25.1 | 1.17 | 2.22 |
| 2 | 1.62 | 0.255 | 25.1 | 1.09 | 2.14 |
| 3 | 5.69 | 0.255 | 25.1 | 5.17 | 6.22 |
| 4 | 1.84 | 0.255 | 25.1 | 1.31 | 2.36 |

Estimate Marginal Means - Training

| | | | | 95% Confidence Intervals | |
|---|---|---|---|---|---|
| Training | Mean | SE | df | Lower | Upper |
| ET | 2.74 | 0.226 | 16.0 | 2.26 | 3.22 |
| HIIT | 2.68 | 0.226 | 16.0 | 2.20 | 3.16 |

## Results Plots

**Plot: LF/HF ~ Timepoint ✻ Training**

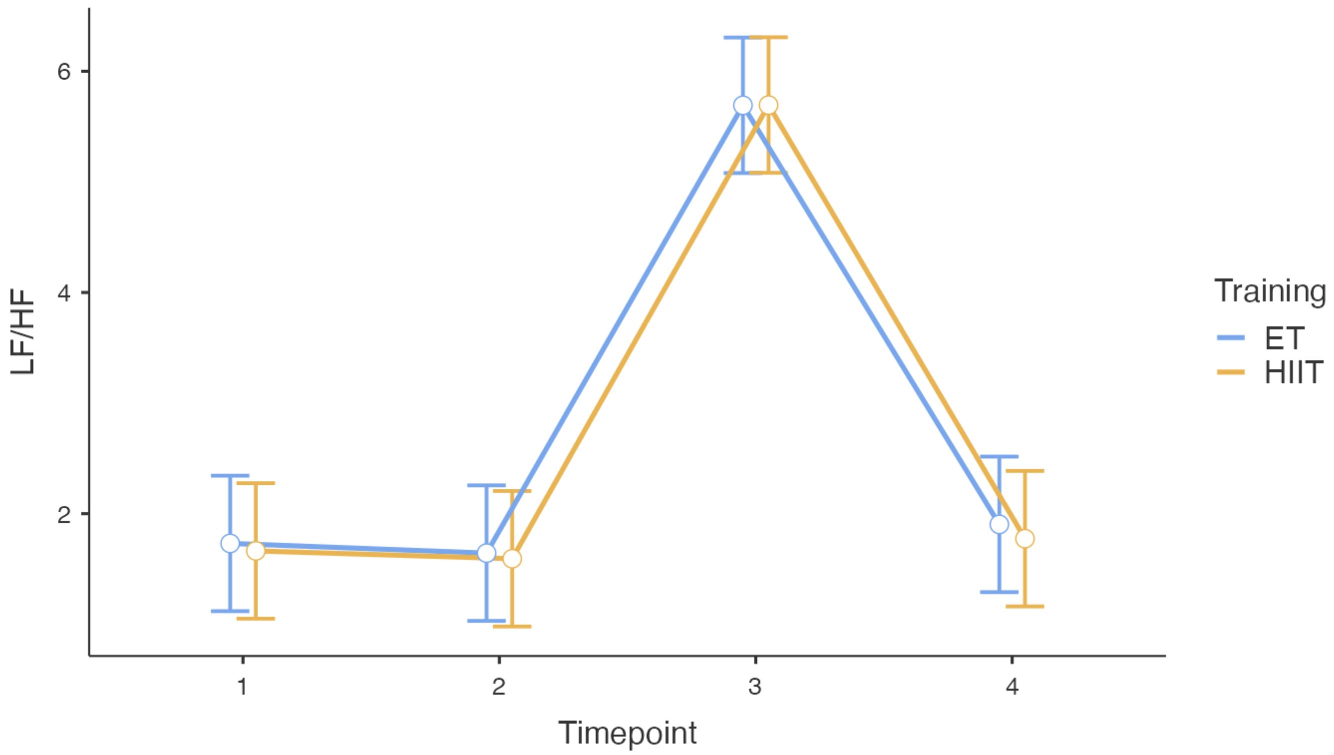


## Assumption Checks

Test for Normality of residuals

| Test | Statistics | p |
|---|---|---|
| Kolmogorov-Smirnov | 0.0681 | .720 |
| Shapiro-Wilk | 0.9827 | .195 |

### Q-Q Plot

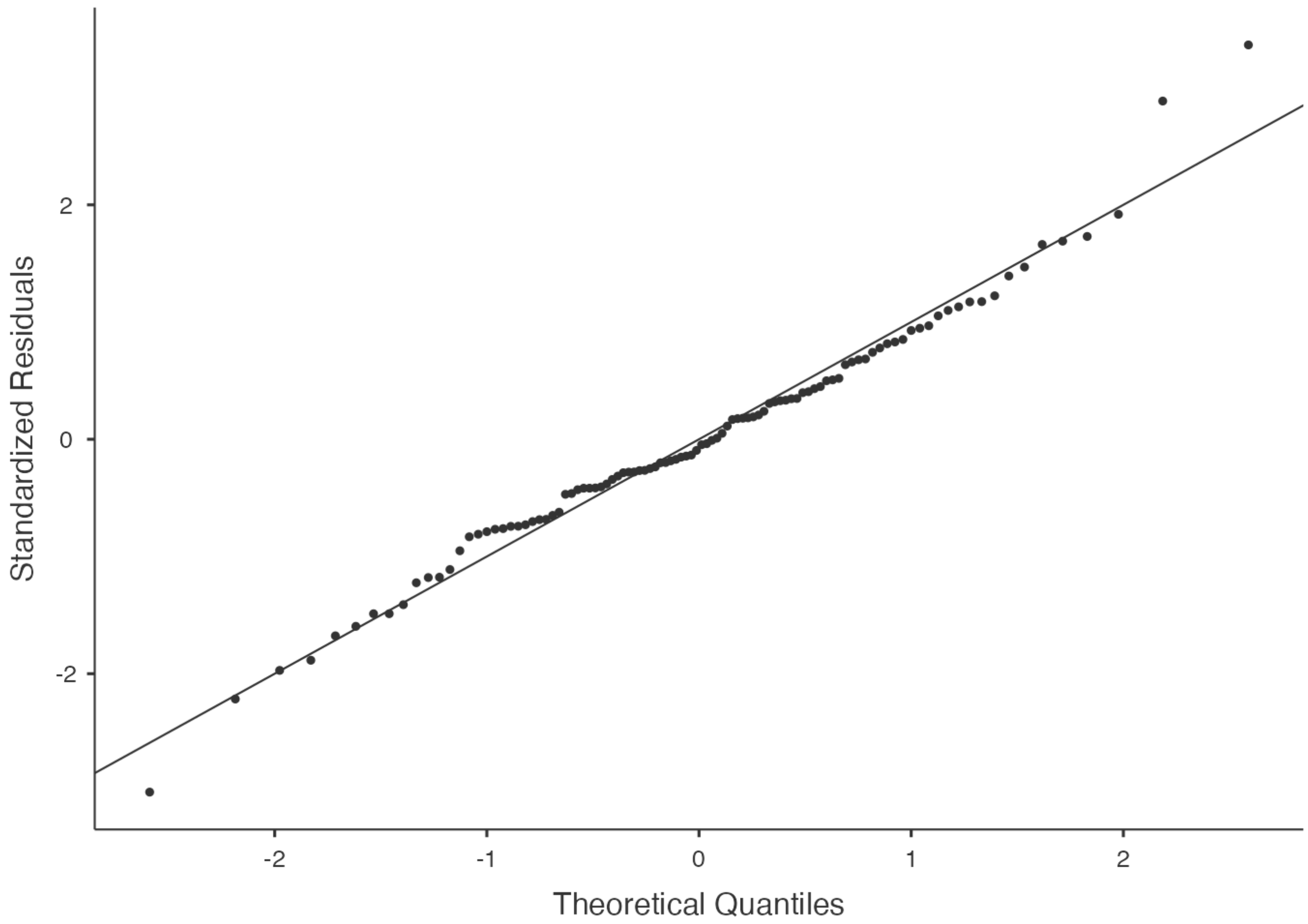


## Mixed Model

Model Info

| Info | | |
|---|---|---|
| Model Type | Mixed Model | Linear Mixed model for continuous y |
| Model | lmer | HR ~ 1 + Timepoint + Training + Timepoint:Training + ( 1 \| ID ) |
| Distribution | Gaussian | Normal distribution of residuals |
| Direction | y | Dependend variable scores |
| Optimizer | bobyqa | |
| DF method | Kenward-Roger | |
| Sample size | 104 | |
| Converged | yes | |
| Y transform | none | |
| C.I. method | Wald | |

**[3]**

## Model Results

Model Fit

| Type | $R^2$ | df | LRT $X^2$ | p |
|---|---|---|---|---|
| Conditional | 0.993 | 8 | 511.284 | <.001 |
| Marginal | 0.991 | 7 | 511.284 | <.001 |

**[4]**

Fixed Effects Omnibus Tests

| | F | df | df (res) | p |
|---|---|---|---|---|
| Timepoint | 4973.270 | 3 | 84.0 | <.001 |
| Training | 2.376 | 1 | 84.0 | .127 |
| Timepoint ✻ Training | 0.234 | 3 | 84.0 | .872 |

Parameter Estimates (Fixed coefficients)

| | | | | 95% Confidence Intervals | | | | |
|---|---|---|---|---|---|---|---|---|
| Names | Effect | Estimate | SE | Lower | Upper | df | t | p |
| (Intercept) | (Intercept) | 84.029 | 0.835 | 82.372 | 85.69 | 12.0 | 100.6824 | <.001 |
| Timepoint1 | 2 - 1 | 0.808 | 1.358 | -1.890 | 3.50 | 84.0 | 0.5946 | .554 |
| Timepoint2 | 3 - 1 | 136.885 | 1.358 | 134.187 | 139.58 | 84.0 | 100.7650 | <.001 |
| Timepoint3 | 4 - 1 | 3.500 | 1.358 | 0.803 | 6.20 | 84.0 | 2.5765 | .012 |
| Training1 | HIIT - ET | 1.481 | 0.961 | -0.426 | 3.39 | 84.0 | 1.5415 | .127 |
| Timepoint1 ✻ Training1 | (2 - 1) ✻ (HIIT - ET) | 2.077 | 2.717 | -3.318 | 7.47 | 84.0 | 0.7644 | .447 |
| Timepoint2 ✻ Training1 | (3 - 1) ✻ (HIIT - ET) | 0.846 | 2.717 | -4.548 | 6.24 | 84.0 | 0.3114 | .756 |
| Timepoint3 ✻ Training1 | (4 - 1) ✻ (HIIT - ET) | 0.231 | 2.717 | -5.164 | 5.63 | 84.0 | 0.0849 | .933 |

**[5]**

Random Components

| Groups | Name | Variance | SD | ICC |
|---|---|---|---|---|
| ID | (Intercept) | 6.06 | 2.46 | 0.202 |
| Residual | | 23.99 | 4.90 | |

Note. Number of Obs: 104 , Number of groups: ID 13

## Post Hoc Tests

Post Hoc comparison: Timepoint ✻ Training

| Comparison | | | | | | | | | |
|---|---|---|---|---|---|---|---|---|---|
| Timepoint | Training | vs | Timepoint | Training | Difference | SE | t | df | $p_{bonferroni}$ |
| 1 | ET | - | 1 | HIIT | -0.692 | 1.92 | -0.360 | 84.0 | 1.000 |
| 1 | ET | - | 2 | ET | 0.231 | 1.92 | 0.120 | 84.0 | 1.000 |
| 1 | ET | - | 2 | HIIT | -2.538 | 1.92 | -1.321 | 84.0 | 1.000 |
| 1 | ET | - | 3 | ET | -136.462 | 1.92 | -71.031 | 84.0 | <.001 |
| 1 | ET | - | 3 | HIIT | -138.000 | 1.92 | -71.832 | 84.0 | <.001 |
| 1 | ET | - | 4 | ET | -3.385 | 1.92 | -1.762 | 84.0 | 1.000 |
| 1 | ET | - | 4 | HIIT | -4.308 | 1.92 | -2.242 | 84.0 | .772 |
| 1 | HIIT | - | 2 | ET | 0.923 | 1.92 | 0.480 | 84.0 | 1.000 |
| 1 | HIIT | - | 2 | HIIT | -1.846 | 1.92 | -0.961 | 84.0 | 1.000 |
| 1 | HIIT | - | 3 | ET | -135.769 | 1.92 | -70.671 | 84.0 | <.001 |
| 1 | HIIT | - | 3 | HIIT | -137.308 | 1.92 | -71.472 | 84.0 | <.001 |
| 1 | HIIT | - | 4 | ET | -2.692 | 1.92 | -1.401 | 84.0 | 1.000 |
| 1 | HIIT | - | 4 | HIIT | -3.615 | 1.92 | -1.882 | 84.0 | 1.000 |
| 2 | ET | - | 2 | HIIT | -2.769 | 1.92 | -1.441 | 84.0 | 1.000 |
| 2 | ET | - | 3 | ET | -136.692 | 1.92 | -71.152 | 84.0 | <.001 |
| 2 | ET | - | 3 | HIIT | -138.231 | 1.92 | -71.952 | 84.0 | <.001 |
| 2 | ET | - | 4 | ET | -3.615 | 1.92 | -1.882 | 84.0 | 1.000 |
| 2 | ET | - | 4 | HIIT | -4.538 | 1.92 | -2.362 | 84.0 | .573 |
| 2 | HIIT | - | 3 | ET | -133.923 | 1.92 | -69.710 | 84.0 | <.001 |
| 2 | HIIT | - | 3 | HIIT | -135.462 | 1.92 | -70.511 | 84.0 | <.001 |
| 2 | HIIT | - | 4 | ET | -0.846 | 1.92 | -0.440 | 84.0 | 1.000 |
| 2 | HIIT | - | 4 | HIIT | -1.769 | 1.92 | -0.921 | 84.0 | 1.000 |
| 3 | ET | - | 3 | HIIT | -1.538 | 1.92 | -0.801 | 84.0 | 1.000 |
| 3 | ET | - | 4 | ET | 133.077 | 1.92 | 69.270 | 84.0 | <.001 |
| 3 | ET | - | 4 | HIIT | 132.154 | 1.92 | 68.789 | 84.0 | <.001 |
| 3 | HIIT | - | 4 | ET | 134.615 | 1.92 | 70.070 | 84.0 | <.001 |
| 3 | HIIT | - | 4 | HIIT | 133.692 | 1.92 | 69.590 | 84.0 | <.001 |
| 4 | ET | - | 4 | HIIT | -0.923 | 1.92 | -0.480 | 84.0 | 1.000 |

## Estimated Marginal Means

Estimate Marginal Means - Timepoint ✻ Training

| | | | | | 95% Confidence Intervals | |
|---|---|---|---|---|---|---|
| Timepoint | Training | Mean | SE | df | Lower | Upper |
| 1 | ET | 48.4 | 1.52 | 74.7 | 45.4 | 51.4 |
| 1 | HIIT | 49.1 | 1.52 | 74.7 | 46.0 | 52.1 |
| 2 | ET | 48.2 | 1.52 | 74.7 | 45.1 | 51.2 |
| 2 | HIIT | 50.9 | 1.52 | 74.7 | 47.9 | 54.0 |
| 3 | ET | 184.8 | 1.52 | 74.7 | 181.8 | 187.9 |
| 3 | HIIT | 186.4 | 1.52 | 74.7 | 183.4 | 189.4 |
| 4 | ET | 51.8 | 1.52 | 74.7 | 48.7 | 54.8 |
| 4 | HIIT | 52.7 | 1.52 | 74.7 | 49.7 | 55.7 |

Estimate Marginal Means - Timepoint

| | | | | 95% Confidence Intervals | |
|---|---|---|---|---|---|
| Timepoint | Mean | SE | df | Lower | Upper |
| 1 | 48.7 | 1.18 | 41.8 | 46.4 | 51.1 |
| 2 | 49.5 | 1.18 | 41.8 | 47.2 | 51.9 |
| 3 | 185.6 | 1.18 | 41.8 | 183.2 | 188.0 |
| 4 | 52.2 | 1.18 | 41.8 | 49.9 | 54.6 |

Estimate Marginal Means - Training

| | | | | 95% Confidence Intervals | |
|---|---|---|---|---|---|
| Training | Mean | SE | df | Lower | Upper |
| ET | 83.3 | 0.963 | 20.9 | 81.3 | 85.3 |
| HIIT | 84.8 | 0.963 | 20.9 | 82.8 | 86.8 |

## Results Plots

**Plot: HR ~ Timepoint ✻ Training**

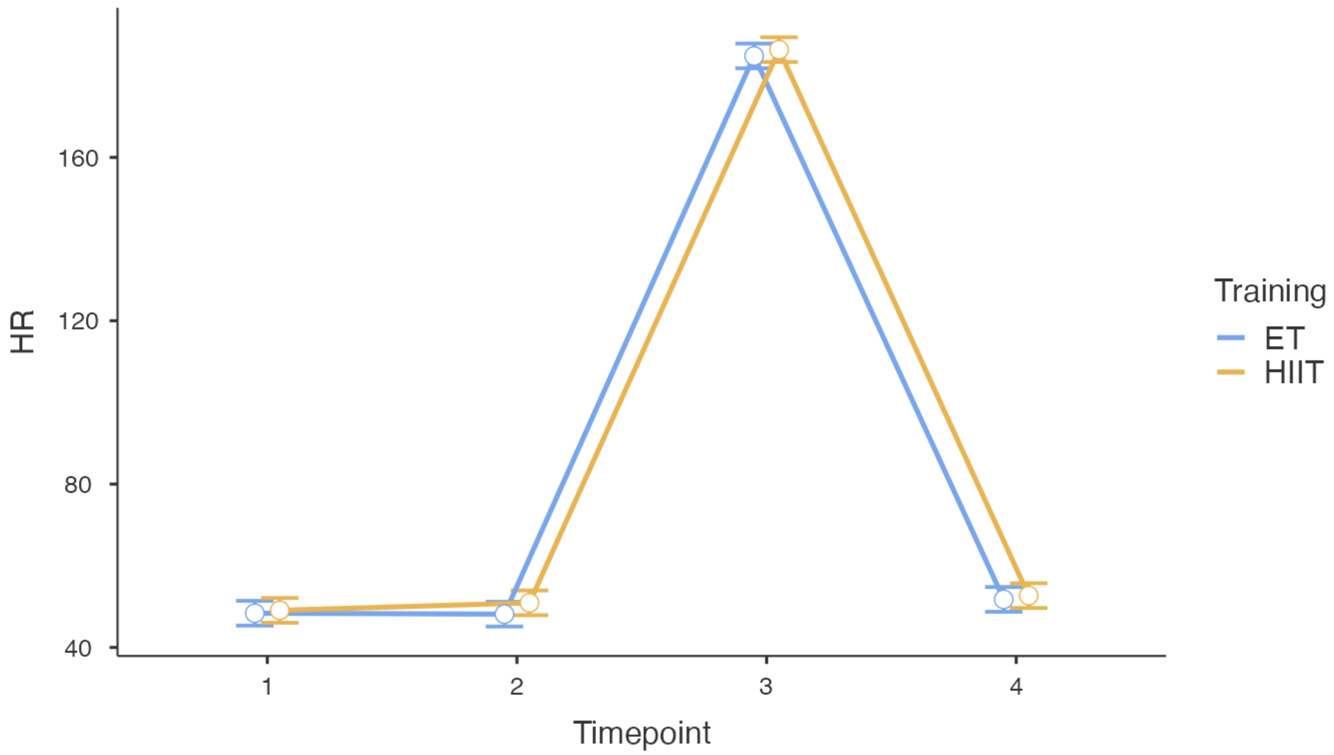


## Assumption Checks

Test for Normality of residuals

| Test | Statistics | p |
|---|---|---|
| Kolmogorov-Smirnov | 0.109 | .168 |
| Shapiro-Wilk | 0.912 | <.001 |

Note. ties should not be present for the one-sample Kolmogorov-Smirnov test

### Q-Q Plot

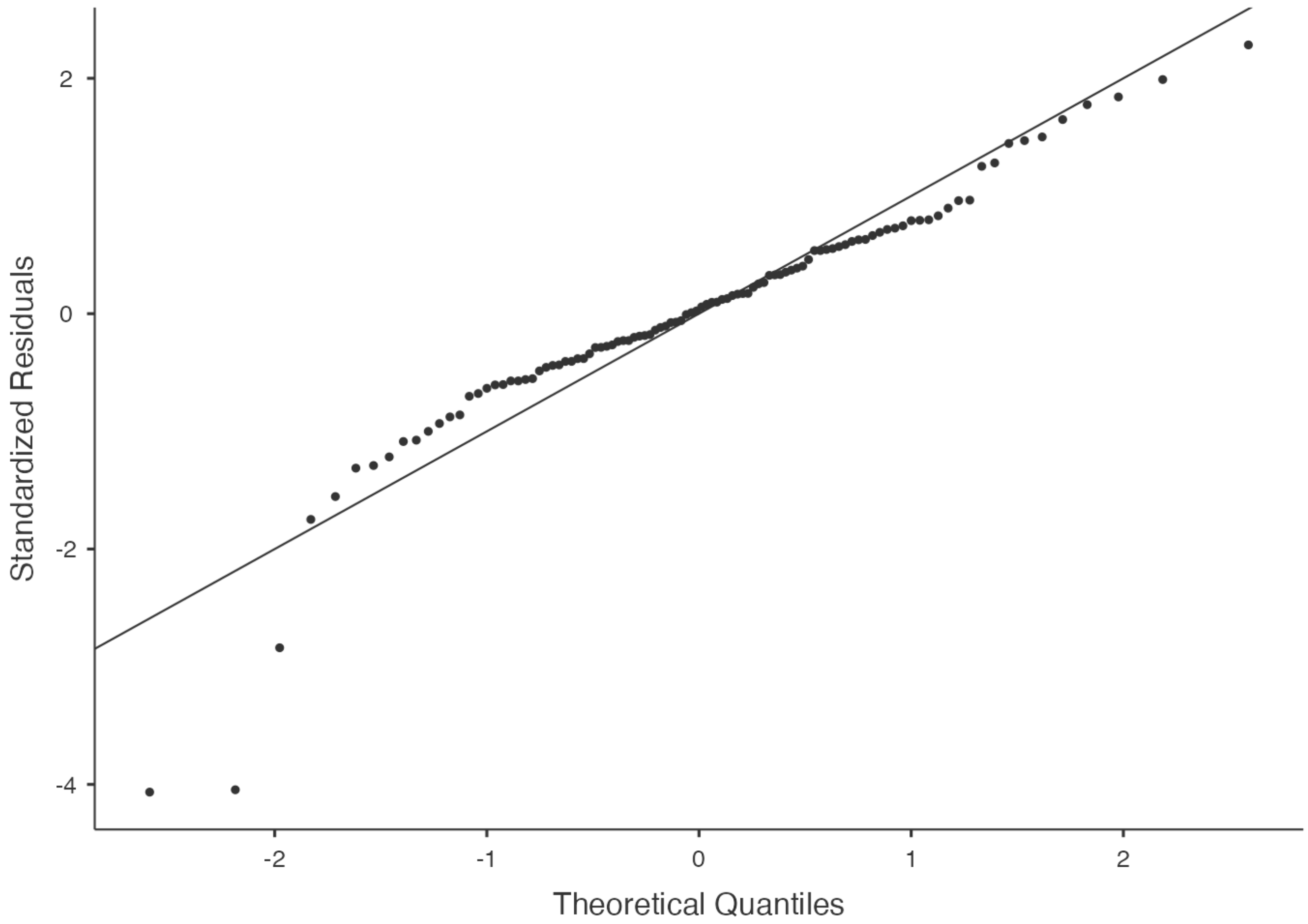


## Descriptives

Descriptives

| | Training | Timepoint | Yaş | Kilo | Boy | HR | HRV | LF/HF | RMSSD | SDNN |
|---|---|---|---|---|---|---|---|---|---|---|
| Mean | ET | 1 | 23.0 | 76.6 | 1.76 | 48.4 | 71.3 | 1.73 | 119 | 179 |
| | | 2 | 23.0 | 76.6 | 1.76 | 48.2 | 70.4 | 1.64 | 116 | 192 |
| | | 3 | 23.0 | 76.6 | 1.76 | 185 | 34.2 | 5.69 | 7.76 | 48.7 |
| | | 4 | 23.0 | 76.6 | 1.76 | 51.8 | 69.5 | 1.90 | 106 | 151 |
| | HIIT | 1 | 23.0 | 76.6 | 1.76 | 49.1 | 71.8 | 1.66 | 108 | 173 |
| | | 2 | 23.0 | 76.6 | 1.76 | 50.9 | 72.2 | 1.59 | 115 | 174 |
| | | 3 | 23.0 | 76.6 | 1.76 | 186 | 30.8 | 5.69 | 6.78 | 30.7 |
| | | 4 | 23.0 | 76.6 | 1.76 | 52.7 | 68.9 | 1.77 | 105 | 138 |
| Std. error mean | ET | 1 | 0.962 | 2.52 | 0.0205 | 0.917 | 0.865 | 0.262 | 4.45 | 10.3 |
| | | 2 | 0.962 | 2.52 | 0.0205 | 0.576 | 0.866 | 0.278 | 5.24 | 6.79 |
| | | 3 | 0.962 | 2.52 | 0.0205 | 3.02 | 1.32 | 0.433 | 0.727 | 3.08 |
| | | 4 | 0.962 | 2.52 | 0.0205 | 0.778 | 1.06 | 0.288 | 4.86 | 12.2 |
| | HIIT | 1 | 0.962 | 2.52 | 0.0205 | 1.00 | 0.724 | 0.211 | 4.84 | 8.17 |
| | | 2 | 0.962 | 2.52 | 0.0205 | 0.746 | 0.668 | 0.242 | 5.26 | 10.0 |
| | | 3 | 0.962 | 2.52 | 0.0205 | 2.23 | 0.769 | 0.393 | 0.439 | 1.50 |
| | | 4 | 0.962 | 2.52 | 0.0205 | 1.03 | 0.828 | 0.258 | 4.38 | 8.39 |
| 95% CI mean lower bound | ET | 1 | 20.9 | 71.1 | 1.72 | 46.4 | 69.4 | 1.16 | 109 | 156 |
| | | 2 | 20.9 | 71.1 | 1.72 | 46.9 | 68.5 | 1.04 | 104 | 177 |
| | | 3 | 20.9 | 71.1 | 1.72 | 178 | 31.3 | 4.75 | 6.17 | 42.0 |
| | | 4 | 20.9 | 71.1 | 1.72 | 50.1 | 67.2 | 1.28 | 95.1 | 124 |
| | HIIT | 1 | 20.9 | 71.1 | 1.72 | 46.9 | 70.3 | 1.20 | 97.3 | 155 |
| | | 2 | 20.9 | 71.1 | 1.72 | 49.3 | 70.7 | 1.06 | 103 | 152 |
| | | 3 | 20.9 | 71.1 | 1.72 | 182 | 29.1 | 4.84 | 5.83 | 27.5 |
| | | 4 | 20.9 | 71.1 | 1.72 | 50.5 | 67.1 | 1.21 | 95.3 | 120 |
| 95% CI mean upper bound | ET | 1 | 25.1 | 82.1 | 1.81 | 50.4 | 73.2 | 2.30 | 129 | 201 |
| | | 2 | 25.1 | 82.1 | 1.81 | 49.4 | 72.3 | 2.25 | 127 | 207 |
| | | 3 | 25.1 | 82.1 | 1.81 | 191 | 37.0 | 6.64 | 9.34 | 55.4 |
| | | 4 | 25.1 | 82.1 | 1.81 | 53.5 | 71.8 | 2.53 | 116 | 177 |
| | HIIT | 1 | 25.1 | 82.1 | 1.81 | 51.3 | 73.4 | 2.12 | 118 | 190 |
| | | 2 | 25.1 | 82.1 | 1.81 | 52.5 | 73.6 | 2.12 | 126 | 196 |
| | | 3 | 25.1 | 82.1 | 1.81 | 191 | 32.4 | 6.55 | 7.74 | 34.0 |
| | | 4 | 25.1 | 82.1 | 1.81 | 54.9 | 70.7 | 2.34 | 114 | 156 |

Note. The CI of the mean assumes sample means follow a t-distribution with N - 1 degrees of freedom

Descriptives

| | | | | | | | | | | |
|---|---|---|---|---|---|---|---|---|---|---|
| Median | ET | 1 | 21.6 | 77.0 | 1.74 | 48 | 71 | 1.68 | 123 | 191 |
| | | 2 | 21.6 | 77.0 | 1.74 | 48 | 71 | 1.57 | 119 | 197 |
| | | 3 | 21.6 | 77.0 | 1.74 | 188 | 34 | 5.38 | 7.61 | 50.1 |
| | | 4 | 21.6 | 77.0 | 1.74 | 51 | 70 | 1.71 | 102 | 154 |
| | HIIT | 1 | 21.6 | 77.0 | 1.74 | 49 | 72 | 1.45 | 104 | 179 |
| | | 2 | 21.6 | 77.0 | 1.74 | 50 | 72 | 1.43 | 117 | 181 |
| | | 3 | 21.6 | 77.0 | 1.74 | 187 | 30 | 5.57 | 7.03 | 28.4 |
| | | 4 | 21.6 | 77.0 | 1.74 | 52 | 68 | 1.43 | 106 | 141 |
| Standard deviation | ET | 1 | 3.47 | 9.08 | 0.0740 | 3.31 | 3.12 | 0.944 | 16.0 | 37.2 |
| | | 2 | 3.47 | 9.08 | 0.0740 | 2.08 | 3.12 | 1.00 | 18.9 | 24.5 |
| | | 3 | 3.47 | 9.08 | 0.0740 | 10.9 | 4.76 | 1.56 | 2.62 | 11.1 |
| | | 4 | 3.47 | 9.08 | 0.0740 | 2.80 | 3.82 | 1.04 | 17.5 | 44.0 |
| | HIIT | 1 | 3.47 | 9.08 | 0.0740 | 3.62 | 2.61 | 0.762 | 17.5 | 29.5 |
| | | 2 | 3.47 | 9.08 | 0.0740 | 2.69 | 2.41 | 0.874 | 19.0 | 36.2 |
| | | 3 | 3.47 | 9.08 | 0.0740 | 8.04 | 2.77 | 1.42 | 1.58 | 5.40 |
| | | 4 | 3.47 | 9.08 | 0.0740 | 3.71 | 2.99 | 0.930 | 15.8 | 30.2 |
| Minimum | ET | 1 | 19.1 | 64.0 | 1.65 | 43 | 67 | 0.590 | 92.8 | 121 |
| | | 2 | 19.1 | 64.0 | 1.65 | 45 | 66 | 0.340 | 90.4 | 146 |
| | | 3 | 19.1 | 64.0 | 1.65 | 162 | 27 | 3.44 | 4.13 | 32.3 |
| | | 4 | 19.1 | 64.0 | 1.65 | 47 | 62 | 0.540 | 82.8 | 88.0 |
| | HIIT | 1 | 19.1 | 64.0 | 1.65 | 43 | 67 | 0.920 | 85.6 | 129 |
| | | 2 | 19.1 | 64.0 | 1.65 | 47 | 69 | 0.340 | 80.8 | 122 |
| | | 3 | 19.1 | 64.0 | 1.65 | 169 | 27 | 3.56 | 4.05 | 24.3 |
| | | 4 | 19.1 | 64.0 | 1.65 | 47 | 65 | 0.610 | 72.8 | 84.0 |
| Maximum | ET | 1 | 29.0 | 91.5 | 1.88 | 56 | 77 | 4.04 | 142 | 228 |
| | | 2 | 29.0 | 91.5 | 1.88 | 52 | 77 | 3.73 | 147 | 243 |
| | | 3 | 29.0 | 91.5 | 1.88 | 196 | 42 | 9.99 | 12.0 | 68.7 |
| | | 4 | 29.0 | 91.5 | 1.88 | 58 | 74 | 4.24 | 128 | 209 |
| | HIIT | 1 | 29.0 | 91.5 | 1.88 | 57 | 76 | 3.85 | 141 | 228 |
| | | 2 | 29.0 | 91.5 | 1.88 | 56 | 76 | 4.02 | 150 | 242 |
| | | 3 | 29.0 | 91.5 | 1.88 | 198 | 36 | 8.02 | 9.12 | 41.1 |
| | | 4 | 29.0 | 91.5 | 1.88 | 60 | 74 | 3.47 | 129 | 181 |
| Skewness | ET | 1 | 0.721 | 0.247 | 0.246 | 0.810 | 0.207 | 1.19 | -0.551 | -0.464 |
| | | 2 | 0.721 | 0.247 | 0.246 | 0.0892 | 0.469 | 0.671 | -0.0920 | 0.145 |
| | | 3 | 0.721 | 0.247 | 0.246 | -1.34 | -0.0525 | 1.76 | 0.151 | 0.328 |
| | | 4 | 0.721 | 0.247 | 0.246 | 0.655 | -0.748 | 1.10 | 0.108 | -0.0400 |
| | HIIT | 1 | 0.721 | 0.247 | 0.246 | 0.501 | 0.0319 | 2.14 | 0.763 | 0.352 |
| | | 2 | 0.721 | 0.247 | 0.246 | 0.369 | 0.0407 | 1.77 | -0.0368 | 0.0149 |
| | | 3 | 0.721 | 0.247 | 0.246 | -0.669 | 0.365 | 0.227 | -0.102 | 0.665 |
| | | 4 | 0.721 | 0.247 | 0.246 | 0.334 | 0.288 | 0.583 | -0.428 | -0.190 |

Note. The CI of the mean assumes sample means follow a t-distribution with N - 1 degrees of freedom

Descriptives

| | | | | | | | | | | |
|---|---|---|---|---|---|---|---|---|---|---|
| Std. error skewness | ET | 1 | 0.616 | 0.616 | 0.616 | 0.616 | 0.616 | 0.616 | 0.616 | 0.616 |
| | | 2 | 0.616 | 0.616 | 0.616 | 0.616 | 0.616 | 0.616 | 0.616 | 0.616 |
| | | 3 | 0.616 | 0.616 | 0.616 | 0.616 | 0.616 | 0.616 | 0.616 | 0.616 |
| | | 4 | 0.616 | 0.616 | 0.616 | 0.616 | 0.616 | 0.616 | 0.616 | 0.616 |
| | HIIT | 1 | 0.616 | 0.616 | 0.616 | 0.616 | 0.616 | 0.616 | 0.616 | 0.616 |
| | | 2 | 0.616 | 0.616 | 0.616 | 0.616 | 0.616 | 0.616 | 0.616 | 0.616 |
| | | 3 | 0.616 | 0.616 | 0.616 | 0.616 | 0.616 | 0.616 | 0.616 | 0.616 |
| | | 4 | 0.616 | 0.616 | 0.616 | 0.616 | 0.616 | 0.616 | 0.616 | 0.616 |
| Kurtosis | ET | 1 | -0.794 | -1.01 | -1.11 | 1.39 | -0.751 | 1.79 | -0.770 | -1.38 |
| | | 2 | -0.794 | -1.01 | -1.11 | -0.512 | 0.0119 | -0.0389 | -1.23 | 0.763 |
| | | 3 | -0.794 | -1.01 | -1.11 | 0.955 | -0.928 | 4.75 | -1.05 | -1.00 |
| | | 4 | -0.794 | -1.01 | -1.11 | 1.07 | -0.0140 | 0.926 | -1.85 | -1.68 |
| | HIIT | 1 | -0.794 | -1.01 | -1.11 | 0.876 | -0.173 | 5.70 | -0.520 | -0.775 |
| | | 2 | -0.794 | -1.01 | -1.11 | -0.652 | -1.15 | 5.00 | -0.126 | -0.521 |
| | | 3 | -0.794 | -1.01 | -1.11 | 0.267 | -0.897 | -0.887 | -0.799 | -0.889 |
| | | 4 | -0.794 | -1.01 | -1.11 | -0.209 | -1.10 | -1.05 | -0.0864 | -0.900 |
| Std. error kurtosis | ET | 1 | 1.19 | 1.19 | 1.19 | 1.19 | 1.19 | 1.19 | 1.19 | 1.19 |
| | | 2 | 1.19 | 1.19 | 1.19 | 1.19 | 1.19 | 1.19 | 1.19 | 1.19 |
| | | 3 | 1.19 | 1.19 | 1.19 | 1.19 | 1.19 | 1.19 | 1.19 | 1.19 |
| | | 4 | 1.19 | 1.19 | 1.19 | 1.19 | 1.19 | 1.19 | 1.19 | 1.19 |
| | HIIT | 1 | 1.19 | 1.19 | 1.19 | 1.19 | 1.19 | 1.19 | 1.19 | 1.19 |
| | | 2 | 1.19 | 1.19 | 1.19 | 1.19 | 1.19 | 1.19 | 1.19 | 1.19 |
| | | 3 | 1.19 | 1.19 | 1.19 | 1.19 | 1.19 | 1.19 | 1.19 | 1.19 |
| | | 4 | 1.19 | 1.19 | 1.19 | 1.19 | 1.19 | 1.19 | 1.19 | 1.19 |
| Shapiro-Wilk W | ET | 1 | 0.891 | 0.939 | 0.944 | 0.944 | 0.961 | 0.913 | 0.918 | 0.890 |
| | | 2 | 0.891 | 0.939 | 0.944 | 0.942 | 0.944 | 0.954 | 0.922 | 0.966 |
| | | 3 | 0.891 | 0.939 | 0.944 | 0.829 | 0.959 | 0.833 | 0.952 | 0.941 |
| | | 4 | 0.891 | 0.939 | 0.944 | 0.963 | 0.915 | 0.907 | 0.872 | 0.904 |
| | HIIT | 1 | 0.891 | 0.939 | 0.944 | 0.976 | 0.970 | 0.787 | 0.917 | 0.942 |
| | | 2 | 0.891 | 0.939 | 0.944 | 0.954 | 0.915 | 0.846 | 0.982 | 0.927 |
| | | 3 | 0.891 | 0.939 | 0.944 | 0.956 | 0.940 | 0.967 | 0.964 | 0.903 |
| | | 4 | 0.891 | 0.939 | 0.944 | 0.981 | 0.945 | 0.914 | 0.976 | 0.961 |
| Shapiro-Wilk p | ET | 1 | .100 | .446 | .512 | .504 | .769 | .202 | .238 | .097 |
| | | 2 | .100 | .446 | .512 | .481 | .511 | .667 | .266 | .841 |
| | | 3 | .100 | .446 | .512 | .015 | .741 | .017 | .627 | .466 |
| | | 4 | .100 | .446 | .512 | .800 | .215 | .164 | .056 | .150 |
| | HIIT | 1 | .100 | .446 | .512 | .958 | .893 | .005 | .231 | .487 |
| | | 2 | .100 | .446 | .512 | .653 | .214 | .025 | .987 | .310 |
| | | 3 | .100 | .446 | .512 | .694 | .458 | .857 | .820 | .146 |
| | | 4 | .100 | .446 | .512 | .984 | .521 | .208 | .952 | .769 |

Note. The CI of the mean assumes sample means follow a t-distribution with N - 1 degrees of freedom

# References

**[1]** The jamovi project (2025). *jamovi*. (Version 2.7) [Computer Software]. Retrieved from https://www.jamovi.org.

**[2]** R Core Team (2025). *R: A Language and environment for statistical computing*. (Version 4.5) [Computer software]. Retrieved from https://cran.r-project.org. (R packages retrieved from CRAN snapshot 2025-05-25).

**[3]** Gallucci, M. (2019). *GAMLj: General analyses for linear models (Version 3.6.5)*. [jamovi module]. Retrieved from https://gamlj.github.io/.

**[4]** Gallucci, M. (2020). *Model goodness of fit in GAMLj*. . link.

**[5]** Lüdecke, Ben-Shachar, Patil & Makowski (2020). *Extracting, Computing and Exploring the Parameters of Statistical Models using R*. CRAN. link.
